# Lactic Acid Bacteria as the Green and Safe Food Preservatives: Their Mechanisms, Applications and Prospects

**DOI:** 10.3390/foods15020241

**Published:** 2026-01-09

**Authors:** Yuwei Zhang, Lianrui Li, Xiaoyang Pang, Shuwen Zhang, Yang Liu, Yunna Wang, Ning Xie, Xu Li

**Affiliations:** 1 Key Laboratory of Agro-Products Quality and Safety Control in Storage and Transport Process, Ministry of Agriculture and Rural Affairs, Institute of Food Science and Technology, Chinese Academy of Agricultural Sciences, Beijing 100193, China; zhangyuwei5608@163.com (Y.Z.);; 2 College of Life Science and Technology, Tarim University, Alar 843300, China; lilianrui51@163.com

**Keywords:** lactic acid bacteria (LAB), food preservatives, antimicrobial mechanism, food control, prospects

## Abstract

Microbial contamination of food is a crucial cause of food spoilage and foodborne diseases, posing a severe threat to global public health. Although chemical preservatives are effective, their potential hazards to human health and the environment, coupled with the growing demand for “clean label” products, have driven the search for natural alternatives. Lactic acid bacteria (LAB), recognized as the Generally Recognized as Safe (GRAS) microorganisms, have emerged as the promising bio-preservatives due to their safety, effectiveness, and multifunctionality. This review systematically summarized the core antimicrobial properties of LAB, including their inhibitory spectrum against foodborne pathogens, spoilage microorganisms, viruses, parasites, and their ability to degrade toxic substances such as mycotoxins, pesticides, and heavy metals. Key inhibitory mechanisms of LAB are highlighted, encompassing the production of antimicrobial metabolites, leading to metabolism disruption and cell membrane damage, nutrition and niche competition, quorum-sensing interference, and anti-biofilm formation. Furthermore, recent advances in LAB applications in preserving various food matrices (meat, dairy products, fruits and vegetables, cereals) are integrated, including their roles in enhancing food sensory quality, extending shelf life, and retaining nutritional value. The review also discusses critical factors influencing LAB’s inhibitory activity (medium composition, culture conditions, ionic components, pathway regulator, etc.) and the challenges associated with the application of LAB. Finally, future research directions are outlined, including the novel LAB and metabolites exploration, AI-driven cultural condition optimization, genetic engineering application, nano-encapsulation and active packaging development, and building up the LAB-based cellular factories. In conclusion, LAB and their antimicrobial metabolites hold great promise as green and safe food preservatives. This review is to provide comprehensive theoretical support for the rational improvement and efficient application of LAB-based natural food preservatives, contributing to the development of a safer and more sustainable food processing and preservation systems.

## 1. Introduction

With the rapid development of the food industry and the increasing frequency of global food trade, food spoilage and contamination have become a global public safety and health concern [1,2]. Notably, microbial contamination is the most critical reasons for food spoilage and the spread of foodborne illnesses, as it not only leads to the deterioration of food flavor, texture, and nutrition, but also may induce the production of toxic chemicals and the spread of foodborne pathogens [1,2]. To address these challenges, various methods have been applied in the modern food industry to effectively inhibit harmful microorganisms, extend food shelf life, and ensure food safety, such as high-temperature sterilization, irradiation treatment, vacuum package, drying processing, and adding preservatives [3]. Among them, the addition of preservatives is an important approach to suppress food microbiology growth, and control food spoilage during processing and storage. Thereof, chemical agents are the most common preservatives in the food industry, including nitrates/nitrites, calcium propionate, potassium sorbate, sulfur dioxide, sodium benzoate, propyl gallate, etc. [4,5]. Although chemical preservatives offer advantages of efficiency and economy, improper usage can pose potential health risks [4,5]. For instance, long-term excessive intake of chemical preservatives exerts adverse effects on human health: sodium benzoate and benzoic acid are widely used in sodas and beverages, but hazardous effects may occur when their concentrations greatly exceed permitted limits—consuming extremely large quantities of sodium benzoate can cause nausea, vomiting, abdominal pain, and, in severe circumstances, central nervous system (CNS) depression [6]. Therefore, people’s demand for “clean labels” for food is continuously increasing. In response to consumers’ pursuit of natural and healthy options, the addition of chemical preservatives has become restricted or even prohibited nowadays. Consequently, biological preservatives, including various probiotics and their metabolites, have gained increasing application in food processing due to their compatibility with healthy lifestyles and environmental friendliness [7]. Particularly, probiotics, especially lactic acid bacteria (LAB), with their advantages of safety, effectiveness, and multifunctionality, have emerged as promising alternatives to chemical preservatives [8].

Probiotics, defined as live microorganisms that confer health benefits for host, have been extensively studied and applied in food, medicine, and agriculture fields [8,9]. As a core group of probiotics, LAB are Gram-positive, non-motile, non-spore forming microorganisms that exist as rods or cocci, and they produce lactic acid by fermenting carbohydrates [8,9]. They encompass diverse genera, such as *Aerococcus*, *Carnobacterium*, *Enterococcus*, *Leuconostoc*, *Oenococcus*, *Pediococcus*, *Streptococcus*, *Tetragenococcus*, *Vagococcus*, *Weissella*, *Lactococcus*, *Bifidobacterium*, and *Lactobacillus*, and are classified into homofermentative and heterofermentative types based on their fermentation patterns [8,9]. As is well-known, LAB possess a wide range of well-documented probiotic functions: they can regulate host gut microbiota balance, enhance intestinal barrier function, modulate immune responses, optimize body metabolism, improve fertility properties, and produce bioactive substances such as short-chain fatty acids, exopolysaccharides, and vitamins that contribute to human health [10,11]. Due to these multifaceted benefits, LAB have been widely integrated into various food categories—from traditional fermented foods, like yogurt, cheese, kimchi, and sourdough, to innovative functional products including probiotic drinks, fermented plant-based milks, and fortified snacks [12]. Their ability to improve food nutritional value, texture, and flavor while delivering health benefits as a cornerstone of functional food development.

Beyond their fermentative and nutritional properties, the antimicrobial potential of LAB has emerged as a key research focus. Having both Generally Regarded as Safe (GRAS) and Qualified Presumption of Safety (QPS) status, LAB hold significant value in the food industry for their prominent antimicrobial functions. Specifically, LABs and their metabolites exhibit strong antibacterial activity against various foodborne pathogens and spoilage microorganisms, offering a superior choice for ensuring food safety [13]. Currently, LAB-based bio-preservation is widely utilized in the processing of dairy products [14], meat [2], staple foods [2], and plant-based foods [15]. Antimicrobial LAB have attracted global attention, and the antimicrobial mechanisms and the bio-preservation applications remain a hotspot in the food microbiology and food science area [16]. In 2024, studies related to the antimicrobial activity and food preservation of LAB exceeded 2500, accounting for 18% of food microbiology research and 3% of food science research [16]. Notably, publications focusing on the antimicrobial properties of LAB account for approximately 46% of all LAB-related studies (URL: pmc.ncbi.nlm.nih.gov/search/?term=Lactic+acid+bacteria+&sort=relevance&filter=dates.2024-2025 (accessed on 17 November 2025)), indicating that research of LAB’s antimicrobial activity is a key focus in both scientific research and industrial applications.

Against this background, the main objectives of this review are clearly defined as follows: (1) to systematically summarize the antimicrobial characteristics of LAB, including their antimicrobial spectrum, antimicrobial metabolites, and key regulatory factors of inhibitory activity; (2) to update and integrate the current knowledge on LAB’s antimicrobial capacity and the underlying mechanisms; (3) to synthesize recent advances in the application of antimicrobial LAB in different food matrices; (4) to propose feasible strategies to enhance LAB’s antimicrobial activity. The core focus of this review lies in exploring the regulatory effects of food matrices on LAB’s antimicrobial efficacy, optimizing the antimicrobial effect and usability of LAB as a preservative and addressing the key technical bottlenecks in industrial application. Ultimately, our review aims to provide comprehensive insights for the development of natural food preservatives (LAB and their antimicrobial metabolites) and offer directions for future research on LAB-based bio-preservation.

## 2. LAB as the Starter and Preservatives in Fermentation Food

**Figure 1:**
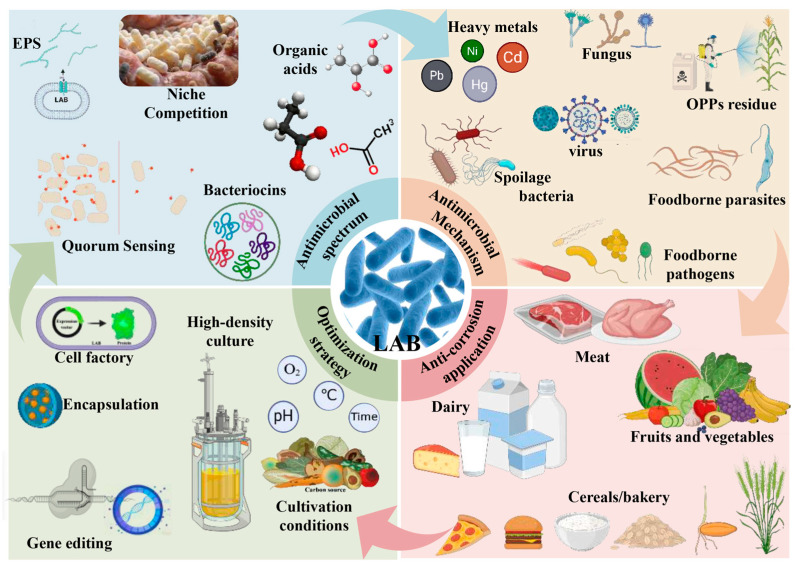
The fermentation and inhibition of LAB on food processing for food safety.

Long-term practical experience shows that naturally fermented foods could be preserved for a long time. Fermentation is an indispensable processing technology in the food industry, which enables preserving food products and prolonging their shelf life, as well as providing the unexpected sensory properties at the same time. Moreover, this processing technology has a favorable impact on the health of intake probiotic microorganisms and extra nutrients in fermentation products [17]. LAB, as the most common fermentative microorganism, are frequently employed, because of the positive contributions to the flavor, texture, and nutritional values in various foods, as well as their natural antimicrobial properties [18]. With a long history of food processing, the fermentative LAB were present in the spontaneous fermentation of different foods, while LAB have been used as the starter in the modern food industry. Nowadays, diverse LAB strains have become widespread in the processing of meat, dairy, vegetables, fruits, and cereal products (Figure 1) (Table 1).

The core roles of LAB in food fermentation and preservation are summarized, including: (1) The fermentation function of LAB in different food matrices (meat, dairy, fruits and vegetables, cereals/bakery); (2) The antimicrobial spectrum of LAB, covering foodborne pathogens, spoilage bacteria, fungi, viruses, foodborne parasites, and their ability to degrade harmful substances (heavy metals, OPPs residues, mycotoxins); (3) Key mechanisms underlying LAB’s antimicrobial activity, including niche competition, quorum sensing interference, production of antimicrobial substances such as bacteriocins, organic acids, and EPS; (4) Strategies to enhance LAB’s application potential (optimized conditions, high-density culture, encapsulation, gene editing, development as cell factories). Symbols and abbreviations: EPS = exopolysaccharides; OPPs = organophosphorus pesticides; Cd = cadmium; Pb = lead; Hg = mercury.

### 2.1. Meat

LAB, as starters in fermented meat, enhance the sensory quality, health benefits, safety, and shelf life of meat products, which can degrade the proteins of meat, impart unique color and flavor, improve texture and taste, promote nutrition digestion, improve absorption, and reduce biogenic amines and nitrites (Table 1) [2]. Li et al. [19] inoculated *Lactiplantibacillus plantarum* H30-2 into fermented surimi and found that LAB promoted the production of flavor compounds such as pentanol, octanol, and nonanol. Zheng et al. [20] reported that inoculating *Latilactobacillus sakei* L.48 into fermented sausages enhanced the formation of flavor substances, including 3-hydroxy-2-butanone, hexanal, and 1-octen-3-ol. *Lactiplantibacillus plantarum*, *Latilactobacillus*. *sakei*, and *Latilactobacillus curvatus* are the most widely used LAB species in meat product fermentation [2,21,22].

LAB have the remarkable capacity of inhibiting spoilage microorganisms and foodborne pathogens in meat products, thereby extending the shelf life of meat products. For instance, *Lactiplantibacillus pentosus* 31-1, isolated from traditionally Chinese fermented Xuan-Wei Ham, was applied as the bio-preservative in chill-stored nonvacuum-tray-packaged pork meat, significantly extending its shelf life [21]. The study demonstrated that *Lactiplantibacillus pentosus* 31-1 could reduce the accumulation of volatile basic nitrogen, and significantly suppressed the growth of *Listeria* and *Pseudomonas* [21]. In another study of Mouafo et al., the biosurfactants produced by *Lacticaseibacillus paracasei* subsp. N2 and *Lacticaseibacillus casei* subsp. TM1b could effectively inhibit the proliferation of *P. aeruginosa* MTCC 1934 and extend the shelf life of raw goat meat up to 15 days [22]. Numerous studies have confirmed the antibacterial efficacy of LAB against psychrophilic bacteria in meat, such as *Pseudomonas* species, underscoring the considerable potential of LAB as natural preservatives in meat and meat products. Yildrim et al. [23] added *Lactococcus lactis* BZ at concentrations from 200 AU/mL to 2500 AU/mL to fresh beef, in which *Lactococcus lactis* BZ produced bacteriocins and exhibited the dose-dependent antibacterial activity. Compared with the control, the inhibition of LAB on psychrophilic bacteria in beef was reduced 3.5 Log after 12 d storage [23].

### 2.2. Dairy

Various LAB naturally exist in raw milk, and diverse dairy products are processed by being spontaneously fermented. In the modern dairy industry, LAB, as starters, are added and used for dairy product fermentation (Table 1), including yogurt, cheese, butter, cream, etc. LAB could modify the state of milk during the fermentation, decompose proteins to promote absorption, and form specific flavor substances, which are more suitable for eating and absorbing [17]. Exopolysaccharides (EPS) produced by LAB are essential to form highly viscous solutions, and their pseudoplastic nature, contributing to texture and rheology of foods, are attributes much appreciated by consumers [24]. Some LAB species, such as nonstarter LAB, also play a fundamental role in the development of aroma and texture during the ripening process of cheeses, contributing to their quality and identity patterns [25]. Furthermore, diacetyl, 2,3-butanedione plays a key role in the distinctive flavor notes in cheeses, butter, and other dairy products, and the compound is synthesized by LAB metabolism of citrate [26].

**Table 1:** LAB applications in different food categories.

Food Category	Specific Food	LAB Strains	Specific Effects	References
Meat	Surimi	*Lactiplantibacillus plantarum* H30-2	Promoted production of flavor compounds	[19]
	Sausages	*Latilactobacillus sakei* L.48	Enhanced formation of flavor substances	[20]
	Chilled pork	*Lactiplantibacillus pentosus* 31-1	Reduced volatile basic nitrogen, inhibited Listeria/Pseudomonas, extended shelf life	[21]
	Raw goat meat	*Lactiplantibacillus paracasei* subsp. N2, *Lactiplantibacillus casei* subsp. TM1b	Produced biosurfactants, inhibited P. aeruginosa, extended shelf life to 15 days	[22]
	Fresh beef	*Lactococcus lactis* BZ	Produced bacteriocins (dose-dependent antibacterial), reduced psychrophilic bacteria by 3.5 Log (12d storage)	[23]
	Cheese	*Lactobacillus acidophilus* ItalTR260	Improved milk texture/flavor, promoted protein absorption; EPS enhanced rheology; nonstarter LAB improved cheese aroma/texture; synthesized diacetyl for flavor	[17,24,25,26,27]
	Yogurt	*Lactobacillus gasseri* BNR17 and *Lactiplantibacillus plantarum* HY7714		[28]
	Cream	*Leuconostoc mesenteroides*	High acid production, antioxidant/antibacterial, inhibited Shigella/Salmonella/Staphylococcus aureus	[29]
Fruits and Vegetables	Sichuan pickles	*Lactiplantibacillus plantarum*, *Levilactobacillus brevis*, *Leuconostoc mesenteroides*	Produced lactic acid (sour taste), promoted flavor substances (aldehydes, esters, etc.)	[30]
	Cucumber pickles	*Levilactobacillus brevis* T7	Improved acceptability, antimicrobial (potential probiotic)	[31]
	Fresh lotus roots	*Lactiplantibacillus plantarum*	Inhibited phenolic oxidation, transformed 84.17% catechin (30 h)	[32]
	Grapes	*Lactiplantibacillus plantarum*	Reduced fungal infection, decreased surface mycotoxin	[33]
	Fresh strawberries	*Lactiplantibacillus plantarum*	Extended shelf life, inhibited yeast/mold growth	[34]
Cereals and Bakery	Cereal products	LAB (non-specific)	Improved amino acid bioavailability, reduced anti-nutrients, enhanced mineral absorption	[15,35]
	Bread	*Lactiplantibacillus plantarum* 5L1	Improved volume/elasticity/flavor, reduced gluten intolerance/glycemic index; inhibited mycotoxin-producing fungi, extended shelf life	[36,37,38]
	Bread (sponge dough)	*Lactococcus lactis* subsp. *diacetylactis*, *Lactobacillus delbrueckii* subsp. *bulgaricus*, and *Lacticaseibacillus rhamnosus*	Extended shelf life by 5 days vs. control	[39]

With the exception of the processing characteristics of LAB, these can inhibit the growth of bacterial contamination in dairy products, effectively ensuring the food safety of fermentation dairy products. *Lactobacillus acidophilus* ItalTR260, isolated from traditional Brazilian cheese, showed antimicrobial effects against pathogenic bacteria, and were considered suitable for improving the quality and functionality of short-aged cheeses [27]. Yang et al. reported that Greek yogurt fermented strain *Lactobacillus gasseri* BNR17 and *Lactiplantibacillus plantarum* HY7714 not only exhibited superior physicochemical properties and higher sensory evaluation scores in yogurt processing, but also could reduce the population of enterohemorrhagic *Escherichia coli* (EHEC), implying their effective inhibitory ability in vitro and in vivo [28]. The LAB species for cream processing *Leuconostoc mesenteroides*, have been confirmed to have a high acid-producing capacity, antioxidant activity, and antibacterial properties. The acidic substances of *Leuconostoc mesenteroides* fermentation showed the significant inhibitory effect on pathogenic bacteria such as *Shigella*, *Salmonella*, and *Staphylococcus aureus* [29].

### 2.3. Fruits and Vegetables

LAB are also commonly used in the processing of plant-based foods, especially green vegetables (Table 1). The fermentation of fruits and vegetables with LAB maximizes the retention of the nutritional value of the raw fruit and vegetable materials. During the fermentation process, various nutrients such as amino acids and short-chain fatty acids are produced [40]. Additionally, fermentation generates abundant probiotic metabolites, which are beneficial for improving the intestinal environment and enhancing human immune function [41]. Sichuan pickles, the most famous fermented vegetable, are fermented by LAB, mainly including *Lactiplantibacillus plantarum*, *Levilactobacillus brevis*, and *Leuconostoc mesenteroides*, which produce lactic acid and endow the characteristic sour taste [30]. LAB also contribute to the generation of aldehydes, esters, ketones, and isothiocyanates, playing an important role in the flavor formation of the pickles [30]. Similarly, the sensory analysis by Guney et al. indicated that LAB-added fermented (*Levilactobacillus brevis* T7) cucumber pickles were more acceptable compared with the spontaneously fermented sample [31]. *Levilactobacillus brevis* T7 demonstrated its antimicrobial activity as potential probiotics, and the cucumber pickles could serve as an alternative probiotic product [31].

In addition to being used for fermentation, LAB are also applied in the preservation of fruits and vegetables. Fresh-cut fruits and vegetables are extremely perishable products with a short shelf life. In a recent study, *Lactiplantibacillus plantarum* suspension was sprayed in fresh lotus roots to limit the oxidation of phenolic compounds to evaluate the postharvest properties of lotus [32]. *Lactiplantibacillus plantarum* suspension proved capable of causing an 84.17% transformation of catechin after interaction for 30 h [32]. For grape storage, Lappa et al. found that LAB effectively reduced the impact of fungal infection on the storage time of grapes, with a significant decrease in mycotoxin production on the grape surface [33]. Additionally, adding *Lactiplantibacillus plantarum* on a fresh strawberry improved the shelf life of the strawberry and reduced the growth of yeast and mold on its surface [34].

### 2.4. Cereals and Bakery

LAB fermentation enables the transformation of the raw material into a new product with unique sensorial properties, new flavors, aromas, better textures, and enhanced nutritional value (Table 1). Cereals, for example, lack the essential amino acids and some vital compounds for proper health, thus LAB fermentation could improve these drawbacks by decreasing the levels of carbohydrates and non-digestible polysaccharides and oligosaccharides, as well as increasing the production and bioavailability of essential amino acids [15]. Non-nutrients, polyphenols, phytates, and tannins were decreased in the process of LAB fermentation, while the binding capacity, digestibility, absorption, and solubility of minerals such as iron were increased [35]. Breads with LAB fermentation exhibited increased volume, elasticity, and flavor, and showed a significant reduction in gluten intolerance and glycemic index, as well as a markedly longer shelf life compared with those without LAB addition [36]. Nowadays, cereal fermented beverages are also gaining great popularity. Rich in active LAB and their metabolites, cereal fermented beverages boasts a variety of nutritional and healthcare functions.

Traditionally, fermented fresh cereal products, such as bread and cereal beverages, have a relatively short shelf life, while the incorporation of LAB fermentation could effectively extend their storage duration. The antibacterial effect of LAB has been widely applied in the processing and preservation of cereal products. For instance, when LAB starters are added to the sponge dough of bread, the shelf life was extended 5 d more than the control [39]. LAB fermentation in bread can inhibit the growth of mycotoxin-producing fungi and remove mycotoxins mainly by binding toxic substances to the cell wall components [37]. When LAB fermentation is incorporated into the processing of traditionally fermented bread, its metabolites, such as lactic acid and phenylpropionic acid, exhibit antifungal activity, which significantly reduces the contamination of *Aspergillus flavus* and *Penicillium verrucosum*, with a notably extended shelf life compared to the previous bread [38].

## 3. Inhibitory Effects of LAB on Foodborne Pathogens and Spoilage Microbes

Food contaminant microorganisms, including foodborne pathogens and spoilage microbes, would not only cause the food contaminations by bacteria, fungi, viruses, parasites, and their toxic derivatives, but also lead to foodborne diseases which severely threaten the health of humans and livestock. In the mass of previous studies, LAB exhibited considerable inhibitory capacity on different harmful microbes, which have been widely used in food processing [13]. In this section, we concluded the inhibition of LAB on different microbes, elaborated the antimicrobial spectrum of LAB, and presented several cases of foodborne microbe control by LAB (Table 2). All the information promotes the application of LAB as natural, safe, and effective food preservatives.

### 3.1. Inhibition of Bacteria

Bacteria are the most critical food contaminators, and 20% of food loss is caused by the spoilage and pathogenic bacteria proliferation in foods. The typical spoilage bacteria, such as *Erwinia*, *Pseudomonas*, *Aeromonas*, *Bacillus*, etc., would proliferate in different foods [2] and destroy the nutrition, flavor, and texture of the food, while other pathogenic bacteria, especially *Salmonella*, *Staphylococcus aureus*, *Enterobacter sakazakii*, *Listeria monocytogenes*, *Bacillus cereus*, etc., could produce bacteriotoxin and cause serious foodborne illnesses in humans [64].

(1) Foodborne pathogens

Foodborne pathogens are the major cause of foodborne diseases and food poisoning, posing a serious threat to human health. Diverse foodborne pathogens commonly contaminate fruits, vegetables, meat, and seafood. Previous studies proved abundant LAB had a wide range of antimicrobial effects against many foodborne pathogens (Figure 1). Almohammadi et al. isolated *Lactiplantibacillus plantarum* LPS10, *Limosilactobacillus fermentum* PP17, and *Pediococcus acidilactici* MH512904 from the pickling process of green olive fruits, and the cell free supernatants (CFS) of all three LAB strains showed apparently inhibitory effects against *Listeria monocytogenes* LMG10470, *Staphylococcus aureus* ATCC25923, *Bacillus cereus* ATCC14579, and *Escherichia coli* ATCC25922, and these strains presented the higher antagonism against Gram^+^ strains than Gram^−^ strains [44]. Similarly, Choi et al. demonstrated that LAB, including *Lacticaseibacillus curvatus* KCCM 43119, *Leuconostoc mesenteroides* KCCM 43060, *Weissella cibaria* KCTC 3746, and *Weissella koreensis* KCCM 41517, could almost completely prevent the growth of four foodborne pathogens (*Salmonella* Enteritidis KCCM 12021, *Salmonella* Typhimurium KCTC 1925, *Staphylococcus aureus* KCCM 11335, and *Escherichia coli* O157:H7 ATCC 35150), increasing the inhibition ratio from 93% to 100% [45]. Dairy products are the resource pool of the antibacterial agent LAB. *Lacticaseibacillus. casei*, isolated from Iranian traditional yogurts, showed obviously inhibitory activity against enteropathogenic *Escherichia coli* and *Salmonella* spp. [65]. Margalho et al. demonstrated that 95.9% of LAB strains isolated from Brazilian artisanal cheeses exhibited antagonistic abilities to *Staphylococcus aureus* and *Listeria monocytogenes* [66]. Additionally, more than direct inhibition, LAB strains also displayed highly co-aggregative activities. LAB could co-aggregate with bacteria, which would lead to the effective inhibition of foodborne pathogenic bacteria adhesion to HT-29 cells, and effective reduction in the harm of foodborne pathogens in the intestine [67].

(2) Spoilage bacteria

Spoilage bacteria on food can cause deleterious effects, such as off odors and color, and shorten the shelf life. Fermented foods usually display a long shelf life, and it is indicated that LAB can be used as natural bio-preservatives, inhibiting the growth of spoilage bacteria. In a study of Mu et al., it was demonstrated that *Lacticaseibacillus paracasei* subsp. *tolerans* N2 and *Lacticaseibacillus*. *casei* subsp. *casei* TM1B, used as bio-preservatives, could suppress 60–80% of *Escherichia coli* in goat meat [22]. In another study, *Lactococcus lactis* subsp. *lactis* PPSSD39 and Weissella cibaria PPSSD19 significantly reduced the severity of *Erwinia mallotivora* infection in papaya plants after 18 days of inoculation, enhancing the biocontrol efficacy to 69% [68]. Psychrophilic bacteria, especially strains of the genus *Pseudomonas*, are the most common spoilers of meat, dairy, vegetables, and fruit in cold-chain logistics, negatively affecting the color, texture, appearance, and flavor of the food [69,70]. According to Zhang et al., inoculation with *Latilactobacillus sakei* and *Latilactobacillus curvatus* significantly reduced the viable colonies of *Enterobacteriaceae*, *Pseudomonas*, and *Bacillus thermoaerophilus* in vacuum-packed chilled beef, and *Latilactobacillus sakei* exhibited a more pronounced inhibitory effect [46]. *Lactiplantibacillus pentosus* 31-1, isolated by Zhang et al., could inhibit 26% the growth of *Pseudomonas fluorescens* at a concentration of 80 AU/mL, significantly extending the shelf life of pork meat [21]. Another study demonstrated that *Lacticaseibacillus paracasei* and *Lacticaseibacillus*. *casei* used as bio-preservatives were able to suppress 64.7% of the growth of *Pseudomonas aeruginosa* [71]. *Brochothrix thermosphacta*, as the thermophilic bacteria in food, is prone to deteriorate meat products, leading to slime formation and sour, musty, or dairy-like odor production. *Latilactobacillus curvatus* and *Latilactobacillus sakei* could not only inhibit *Brochothrix thermosphacta* in vacuum-packaging lamb, with at least 0.5 log reduction, but also displayed the suppression against *Enterobacteriaceae* and *Pseudomonas* spp. [46].

(3) Bacteria biofilm

Biofilms formed by foodborne pathogens and spoilage bacteria are a major challenge in food safety, as they enhance microbial resistance to antibiotics, disinfectants, and environmental stresses, leading to persistent contamination and food spoilage [72]. LAB exhibit significant anti-biofilm effects, which contribute to their antimicrobial activity in food preservation [73]. Studies have shown that LAB exhibit anti-biofilm activity against various foodborne pathogens, including *Listeria monocytogenes*, *Staphylococcus aureus*, *Escherichia coli* O157:H7, and *Pseudomonas aeruginosa* [73]. For example, *Lactiplantibacillus plantarum* LPS10 inhibits the biofilm formation of *Listeria monocytogenes* by 60%. The EPS of *Lactiplantibacillus plantarum* YW32 interferes with the biofilm activity of *Staphylococcus aureus* by inhibiting bacterial adhesion [74]. The cell-free supernatant of different LAB could suppress the biofilm formation and Acetylated homoserine lactones (AHLs) level with a pH-dependent manner [75]. These findings highlight the importance of LAB’s anti-biofilm effects in food preservation, providing an additional mechanism for their antimicrobial activity.

Therefore, LAB, as natural and safe probiotics, could be used as effective alternatives to chemical additives controlling the bacterial contamination in food processing and preservation.

### 3.2. Inhibition of Fungal

Fungi are another important group of food contaminator, including yeasts, molds and their metabolites. Most spoilage fungi disseminate via aerial spores and reproduce through hyphal growth. Their rapid growth and dissemination cause severe food waste. The contamination has significant implications for the food industry and consumer health. Molds decompose proteins, fats, carbohydrates, and vitamins in food, leading to nutrient loss and quality deterioration. Particularly, several toxigenic fungal could synthesize highly toxic chemicals and mycotoxins, which seriously threaten consumer health [76]. Fungicide predominantly consist of highly toxic chemical agents, and there is a lack of safe, efficient, low-toxicity, and naturally fungal inhibitors that are suitable for food processing [7]. LAB can effectively suppress the proliferation of the spoilage fungi through complex and multifaceted mechanisms, representing a promising approach for controlling food spoilage fungi (Table 2).

(1) Yeasts

Yeasts are particularly prevalent in environments with slightly acidic and high sugar conditions, such as fruits and vegetables. *Saccharomyces cerevisiae* is the most notable yeast, which is essential for making bread, beer, and wine. But for some yogurt or fermented beverages, *Saccharomyces cerevisiae* would be one kind of important spoiler [77]. In yogurt processing, *Lacticaseibacillus paracasei* could effectively inhibit the growth of *Saccharomyces cerevisiae* by rapidly producing lactic acid [78]. The application of LAB effectively reduced the number of ethanol-producing yeasts on fresh-eating grapes, and decreased the alcohol content of grapes during storage [79]. *Candida albicans*, focused in medicine, are the opportunistic pathogens. *Limosilactobacillus reuteri* showed antifungal potential, and two *Limosilactobacillus reuteri* strains were observed in co-aggregation and growth inhibition on three different *Candida* species [80]. More research studied the interaction of LAB and yeast in fermentation, to improve the aroma and flavor of wine and Baijiu [81].

(2) Molds

Molds are usually contaminated in grains, nuts, fruits, and meat, severely affecting food quality and economic value. More terribly, several molds can produce mycotoxins, such as the strains of *Aspergillus*, *Penicillium*, and *Fusarium*, whose mycotoxins pose serious health risks to both humans and animals. Ingestion of mycotoxin-contaminated food, which can cause both acute poisoning, including hepatic and renal damage, immune system impairment, and even mortality, and chronic low-dose exposure, which can increase the risk of hepatocellular carcinoma, renal cancer, and other malignancies, would extremely threaten the health of humans and animals [82].

LAB, as GRAS strains worldwide, metabolites such as organic acids (Figure S1), fatty acids, and cyclic dipeptides can inhibit the growth and toxin production of fungi. LAB have been widely used in food and feed to prevent mold contamination. Corsetti et al. reported that *Lactobacillus sanfrancisco* CB1, isolated from sourdough, exhibited inhibitory effects against *Fusarium*, *Penicillium*, *Aspergillus*, and *Monilia*, and identified its antifungal substances, including formic acid, acetic acid, propionic acid, butyric acid, n-valeric acid, and hexanoic acid [83]. Baek et al. found that the organic acids produced by the fermentation of *Leuconostoc citreum* and *Weissella confuse*, including lactic acid and butyric acid, could effectively delay the growth of *Cladosporium* sp. YS1, *Neurospora* sp. YS3, and *Penicillium crustosum* YS2 in rice dough. Moreover, the antifungal activity of the LAB in rice dough was far superior to that of 0.3% calcium propionate [84]. Cheese is also frequently contaminated by molds. Sedaghat H. et al. demonstrated that five non-fermenting LAB strains (*Lactiplantibacillus plantarum* PIN, *Lactiplantibacillus plantarum* CAG23, *Lacticaseibacillus*. *casei* D31, *Lactiplantibacillus pentosus* H39, and *Lactiplantibacillus plantarum* NBRC107151) were all capable of delaying the mycelial growth of *Aspergillus flavus* and *Aspergillus parasiticus* on the cheese surface. Among these strains, *Lactiplantibacillus plantarum* PIN showed the strongest antifungal activity [85]. In citrus, Chen et al. controlled *Penicillium digitatum* infection with diverse LAB strains, including *Lactiplantibacillus plantarum*, *Lactobacillus parafarraginis*, *Lacticaseibacillus casei*, *Lacticaseibacillus paracasei*, *Lactobacillus. buchneri*, and *Weissella paramesenteroides*, and LAB strains could inhibit the growth of *Penicillium digitatum* in citrus with varying degrees [42]. The antifungal activity of LAB has also been widely applied in silage. In 2009, Tanaka et al. inoculated *Lactobacillus coryniformis* strain 394 into silage and found that this strain produced 3-hydroxypropionaldehyde during fermentation, which effectively prevented mold spoilage and aerobic deterioration of the silage [86]. In addition, the direct application of metabolites produced by LAB can also effectively inhibit fungal contamination. Black et al. added 0.15% coriolic acid, fermented with *Lactobacillus hammesii*, during the production of bread, which effectively inhibited the growth of the spoilage *Aspergillus aculeatus*, and extended the shelf life of the fermented bread by 6 days [87].

Further, detoxification of mycotoxins by LAB appears more practical and economical. LAB has two mechanisms for the detoxification of mycotoxins from foods. Food detoxification is achieved with the absorption of the cell wall of LAB, and/or is achieved with the degrading enzymes produced by specific LAB strains [88,89,90]. *Lactobacillus* possess cell walls characterized by dense layers of peptidoglycan, teichoic acid, polysaccharides, and surface proteins. This highlights the unique architecture and composition of the *Lactobacillus* cell wall, which could effectively adsorb and neutralize mycotoxins [88]. According to Chlebicz et al., the cell wall peptidoglycans, polysaccharides, and teichoic acids of *Lactobacillus* bind to aflatoxin B_1_ (AFB_1_) mainly through hydrophobic interactions [88]. The stability of the LAB-AFT complex is an important indicator for evaluating the effectiveness of LAB as a detoxicant. Martínez et al. found that *Lacticaseibacillus rhamnosus* RC007 can adsorb 61% of aflatoxin M_1_ (AFM_1_) in milk, and the formed complex exhibits a certain degree of stability [89]. Furthermore, LAB utilize their enzymes to degrade mycotoxins. Oxidoreductase produced by LAB are vital in transforming mycotoxins into safe or less-toxic catabolites [90]. Zheng et al. reported that *Lacticaseibacillus. casei* YZU01 could produce an extracellular enzyme with high-efficiency patulin (PAT)-degrading activity when induced by patulin, and this enzyme plays a key role in bacterial elimination of patulin [90]. Escrivá et al. reported that aflatoxin B1 and ochratoxin A were reduced by *Lactiplantibacillus plantarum* during bread-making, with degradation rates of 27% and 32%, respectively [57]. Similarly, Murtaza et al. found that *Lactobacillus acidophilus*, *Lactiplantibacillus plantarum* L1, *Lactiplantibacillus plantarum* L2, and *Lacticaseibacillus paracasei* degraded ZEN by 86.14% to 90.39% [58].

With the increasing research and application of LAB in food and feed, their multiple functions on contaminated fungi have been continuously explored. As an effective alternative to chemical additives for fungal inhibition, LAB hold significant application value in the food industry.

### 3.3. Inhibition of the Virus

LAB are also evolving as a novel wave of antagonists against certain foodborne viruses. Although viruses cannot replicate in food, the contamination and ingestion of foodborne viruses may lead to systemic infections. From an epidemiological point of view, human noroviruses (NOVs), rotaviruses, and hepatitis A viruses are foodborne viruses with significant health concerns.

LAB would exhibit antiviral ability by inhibiting the viral life cycle. Lange-Starke et al. showed that the cell-free supernatant of *Latilactobacillus curvatus*, used in sausage processing, inhibited a 1.25 log decrease in the titer of murine NOV S99, substitution of human NOV, was also decreased compared than control [50]. Similarly, Mokoena et al. [51] demonstrated that both culture filtrate and cell suspensions of *Lactococcus lactis* subsp. *lactis* LM0230 significantly inhibited feline calicivirus. The EPS of LAB could also inhibit the virus. The EPS of *Lactiplantibacillus plantarum* LRCC5310, isolated from the Korean traditional fermented food kimchi, significantly suppressed human rotavirus replication by approximately 41.3% both in vitro and in vivo [52]. It is suggested that the antivirus mechanisms of LAB would be (1) denaturation of capsid proteins by the lower pH; trapping viral particles with the peptidoglycan structure of LAB; (3) prevention of the entrance of the virus into the host by diverse metabolites of LAB, such as bacteriocins and hydrogen peroxide; (4) the competition for attachment site of host cells between bacterial cells and virus [91]. In addition, LAB would exhibit antiviral ability by the enhancement of human immunity metabolites. The induction of LAB on host cells to produce reactive oxygen substances might kill several viruses. LAB further displayed therapeutic activity. Watanabe et al. determined that a dispersed LAB (d-LAB), homogenized with a high-pressure homogenizer, inhibited approximately 88.2% of influenza A virus (A/NWS/33, H1N1 subtype) in bronchoalveolar lavage fluid and about 68.8% in lung samples [49]. However, there are no studies that demonstrate LAB directly inhibit foodborne viruses in food matrices. All above findings collectively indicated the considerable abilities of LAB in antiviral applications.

### 3.4. Inhibition of Foodborne Parasites

At present, diseases caused by foodborne parasites are becoming increasingly prevalent. Human infection occurs upon ingestion of undercooked or raw meat contaminated with the infective larvae of a parasite, which primarily affects the gastrointestinal tract, followed by systemic dissemination of the larvae to the muscles, resulting in symptoms such as myalgia, fever, and, in severe cases, cardio pulmonary complications [92]. Currently, foodborne parasites mainly include *Trichinella spiralis*, *Trypanosoma brucei*, *Trypanosoma cruzi*, etc., and the antiparasitic properties of probiotics have also garnered increasing attention [93].

Several studies have indicated that LAB also possessed the potential to prevent and treat parasitic diseases. Shanawany et al. demonstrated that being treated with *Levilactobacillus brevis* (10^9^ CFU/mL/animal), the model mice, infected by *Trichinella spiralis*, were alleviated of intestinal and muscle damage, which contributed to the restoration of the tissue-barrier integrity [56]. Further, the inhibitions of LAB were against both adult and larval *Trichinella spiralis* exceeded 91% [56]. Rahimi et al. showed that lactic acid obviously inhibited *Sarcocystis bradyzoites* in meat, which could be the critical factor of LAB inhibition on parasites [94]. In studies on the prevention or treatment of parasitic diseases, *Enterococcus faecalis* UGRA10 is one of the most well-known and extensively investigated strains, due to its ability to produce a bacteriocin, circular enterocin AS-48 [53,54]. Martín-Escolano et al., in a study on Chagas disease caused by parasite *Trypanosoma cruzi*, found that the rates of infected cells decreased with increasing concentrations of AS-48, with an IC_50_/72 h of 2.62 μM, which was 6-fold lower than that for Benznidazole [53]. The trypanocidal activity of bacteriocin AS-48 can be explained in a mitochondrion-dependent manner by reactive oxygen production and mitochondrial depolarization [53]. At present, relatively few LAB have been found to effectively inhibit parasites, but LAB have the advantages of safety and edibility. In addition, the abundant metabolites produced by LAB also provide a compound pool for parasite inhibition (Table 2).

### 3.5. Biodegradation of Toxic Chemicals and Their Derivatives

The chemical pollutants in food also could be eliminated by LAB. Food chemical pollutants, such as pesticide residues and heavy metals, are the most critical chemical in food, and are involved in food safety incidents [63]. Organophosphorus pesticides (OPPs) are a group of common residual contaminants in food, which cause serious harm to human health. Yuan et al. [63] showed that LAB strains, including *Lactobacillus acidophilus* CICC20244, *Limosilactobacillus reuteri* CICC23151, *Lactiplantibacillus plantarum* CICC20261, *Bifidobacterium animalis* CICC21717, *Lactobacillus helveticus* CICC6032, and *Lactobacillus. delbrueckii* CICC6047, exhibited potential protective effects against pesticide toxicity, which upon the addition of OPPs in the medium, these LAB strains displayed the OPP degradation capabilities, with degradation rates ranging from 2.11% to 87.34%. Among them, *Lactiplantibacillus plantarum* showed the highest degradation rates for four different OPPs (dimethoate: 87.34%, trichlorfon: 76.46%, chlorpyrifos: 83.41%, parathion methyl: 70.63%) [63].

LAB have also been confirmed to have the bio-removal capacities for heavy metals (HM). Heavy metals have been dispersed by farming and irrigating, and ultimately permeated crops and food products, threatening consumer health [95]. In the investigation of Sanjabi et al., LAB, including *Lacticaseibacillus casei*, *Lacticaseibacillus rhamnosus*, *Lactiplantibacillus plantarum*, *Limosilactobacillus fermentum*, *Lactobacillus helveticus*, and *Lactobacillus acidophilus*, could bind lead, cadmium, and nickel in solutions, of which the removal rates ranged from 47.65% to 79.75% for lead, 22.27% to 75.28% for cadmium, and 67.72% to 83.99% for nickel [62]. *Weissella viridescens* is another bioactive LAB. *Weissella viridescens* ZY-6 showed strong binding capacity at the low cadmium concentrations, of which its removal rate was nearly 100% at a cadmium concentration of 10 mg/L [61].

Diverse LAB strains could be used as effective bio-degraders and biosorbents for chemical pollutants in the environment, human diets, and animal feed. Therefore, due to their detoxification properties, the unique architecture and composition of the *Lactobacillus* cell wall fundamentally underlies the capacity to effectively adsorb and neutralize toxic chemicals.

## 4. Antimicrobial Metabolites and the Inhibitory Mechanism of LAB

**Figure 2:**
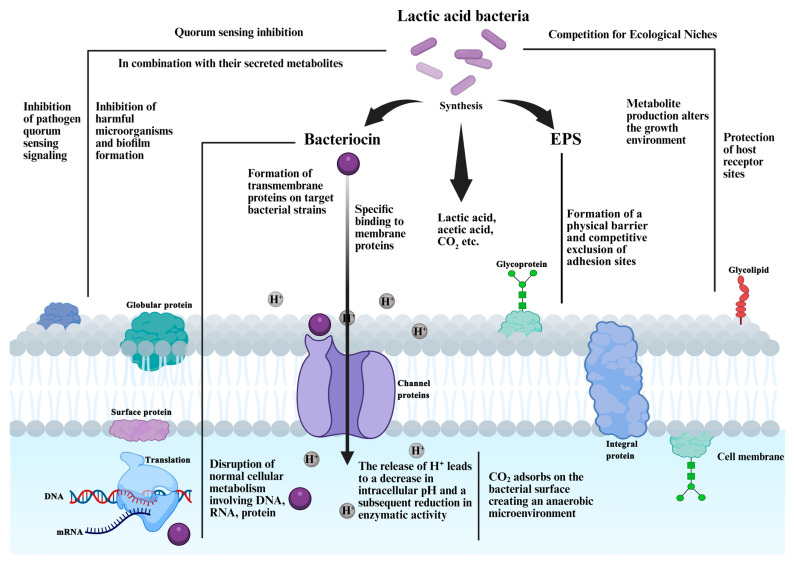
The inhibitory mechanism of LAB on foodborne microorganisms.

In recent years, there has been growing interest in the antimicrobial properties of LAB, and the inhibitory mechanisms also have been revealed by numerous studies. In brief, LAB could reduce the microenvironment pH, produce various antimicrobial secondary metabolites, such as bacteriocins, organic acids, hydrogen peroxide, and exopolysaccharides (Figure 1), compete for the nutrition and ecological niche, and vary the microflora by signaling adjustment, as well as change the food microenvironment. In this section, we summarize the key antibacterial substances produced by LAB and elucidate the mechanisms by which these substances inhibit other microorganisms (Figure 2), thereby providing comprehensive theoretical support for enhancing and applying the antibacterial activity of LAB.

This figure illustrates the multiple pathways of LAB inhibition on foodborne microorganisms: (1) Acid production: LAB metabolize carbohydrates to produce acidic compounds (e.g., lactic acid, acetic acid, CO_2_), which reduce the extracellular pH. H^+^ penetrates the cell membrane of target microorganisms, decreasing intracellular pH and inactivating key metabolic enzymes; CO_2_ adsorbs on the bacterial surface, creating an anaerobic environment that inhibits aerobic microorganisms. (2) Bacteriocin production: LAB synthesize bacteriocins that specifically bind to membrane proteins or form transmembrane pores on target bacterial strains, disrupting cell membrane integrity and inhibiting normal cellular metabolism (DNA, RNA, and protein synthesis). (3) Exopolysaccharide (EPS) production: EPS forms a physical barrier, competitively excluding adhesion sites on host cells, and interferes with the biofilm formation of harmful microorganisms. (4) Quorum sensing (QS) inhibition: LAB interfere with the QS signaling of pathogens by producing QS inhibitors, altering the expression of QS-regulated genes (e.g., virulence factors, biofilm formation). (5) Competition for ecological niches and nutrients: LAB compete with harmful microorganisms for limited nutrients (e.g., carbohydrates, amino acids, iron) and adhesion sites, suppressing their growth and colonization. Symbols and abbreviations: EPS = exopolysaccharides; QS = quorum sensing.

### 4.1. Acid-Producing

LAB metabolize carbohydrates to produce substantial amounts of organic acids, such as lactic acid, thereby reducing the environmental pH to 3.5–4.5. Mauch et al. noted that the inhibition was caused due to the culture supernatant of *Levilactobacillus brevis* PS1 was maximum at pH < 4 [96]. The acidic condition effectively inhibits the growth of various common spoilage and pathogenic microorganisms. For instance, most neutral-preference bacteria, including many strains of *Escherichia coli* and *Salmonella*, have inhibited growth at pH < 4.5. Notably, even acid-tolerant bacteria like *Staphylococcus aureus* are significantly suppressed at pH < 5.0 [97,98]. The primary mechanism involves the penetration of H^+^ into the cell membrane, leading directly to a decrease in the intracellular pH, in which this acidification causes denaturation and inactivation of key enzymes essential for vital metabolic processes [99] (Figure 2). In contrast, LAB maintain a growth advantage under low-pH conditions through adaptive mechanisms such as the expression of acid resistance systems and membrane modifications [99].

More than low-pH, numerous organic acids themselves also possess the ability of bacterial inhibition. With the special growth and metabolism characters, LAB can produce various acidic substances, primarily including lactic acid, citric acid, acetic acid, phenyllactic acid, propionic acid, etc. (Table 3, Figure S1) [100]. The specific products would be varied depending on the different substrate. In a study by Zalán et al., the production of organic acids by 10 LAB strains was determined under different conditions. All strains produced organic acid in the largest concentration in MRS broth, with lactic acid 400–851 mM and acetic acid 25–150 mM [101]. Ji et al. found that the succinic acid content in the fermentation juice by *Limosilactobacillus reuteri*, with the highest value of 130.214 mg/L [102]. Differential organic acids display the remarkable inhibitory effect on food polluted by microorganisms. Acetate, formate, and lactate (0.1 M) produced by LAB, could completely inactivate the proliferation of *Bacillus cereus* at pH 6.4, 6.0, and 5.6, respectively, suggesting organic acids would suppress microorganism independently on H^+^ [103]. Further, spores of *Bacillus cereus* were more resistant to these organic acids than vegetative cells, but 0.1 M formate, lactate, and acetate also showed 50% inhibition of spore germination, displaying the inhibition of organic acid on resistant spores [103].

Both lactic acid and acetic acid are the two major organic acids, which can partially dissociate in aqueous solution to release H^+^. However, at the same concentration, lactic acid exhibits stronger antimicrobial activity. Similarly, acid molecules can also cause cell membrane rupture. The undissociated acid molecules are biologically active, because their lipophilic and traversing cytoplasmic membrane of bacteria [104]. Due to the higher pH, the acid dissociates and releases protons and anions (conjugate bases), thereby disrupting the membrane proton motive force. The accumulation of protons acidifies the cytoplasm, inhibiting cellular metabolic activities and leading to reduced ATP production [105]. Phenyllactic Acid (PLA) is another organic acid produced by specific LAB strains, which has been proven to affect the growth and metabolism of foodborne pathogenic bacteria and fungi. PLA harbors broad spectrum antibacterial activity, inhibiting the growth of microbes and destroying the biofilm structures of bacteria and fungi [106]. Lavermicocca et al. separated PLA from the cell-free supernatant of *Lactiplantibacillus plantarum* FST1.7, which showed antifungal activity [107].

LAB also metabolize several small acidic molecules, such as hydrogen peroxide (H_2_O_2_) and carbon dioxide (CO_2_). Production of H_2_O_2_ by LAB can prevent the growth of foodborne pathogens (Table 3). H_2_O_2_ plunders electrons and molecules of nearby microorganisms, and thus sterilizes by destroying protein molecular structure. Martin and Maris proved that tested the synergistic effect of H_2_O_2_ and 17 kinds of acids on fungi, resulting in stronger antifungal activity [108]. In addition to organic acids, CO_2_ is a major end-product of heterolactic fermentation by LAB, in which it can create a low-oxygen or anaerobic environment (Figure 2) and dissolve in water to form carbonic acid, thereby exerting certain inhibitory effects on some Gram-negative aerobic bacteria and molds [109].

**Table 3:** Principal inhibitory substances produced by LAB.

Inhibitory Substance		Types	Inhibitory Mechanism	Inhibitory Effect	References
Organic acid		lactic acid	Intracellular acidosis and anion accumulation, disrupt the cell membrane	*Ligilactobacillus agilis* ZY25 and *Ligilactobacillus salivarius* ZY35, verified by in vitro antibacterial experiments	[110,111]
		citric acid	Interfere with the activity of key metabolic enzymes, disrupt energy metabolism		
		amber acid			
		malic acid			
		acetic acid	Intracellular acidosis and anion accumulation		
		propionic acid		*Latilactobacillus curvatus* CCFM1268, verified by in vitro antibacterial experiments	[112,113]
		butyric acid			
		formic acid	Inhibit the activity of DNA polymerase; Block the DNA replication	*Lactococcus lactis*, *Lactococcus lactis cremoris*, *Enterococci durans*, *Enterococci faecalis*, verified by in vitro antibacterial experiments	[114,115]
		pyruvicacid	Interfere with the activity of key metabolic enzymes, disrupt energy metabolism	*Enterococcus faecium*, verified by in vitro antibacterial experiments	[116,117]
		benzoic acid	Acidify the cytoplasm, inhibit mitochondrial respiration	*Lactobacillus acidophilus* La-5, verified by in vitro antibacterial experiments	[118,119]
		sorbic acid	disrupt the cell membrane		
		chlorogenic acid	Disrupting the cell membrane, chelating with metal ions, inhibiting enzyme activity, and inducing oxidative stress	*Lacticaseibacillus casei* H1, verified by in vitro antibacterial experiments	[120,121]
Bacteriocin	Class I	nisinF; nisin Q; nisin Z	Damage to cell membrane integrity, specific target Lipid II	*Lactococcus lactis* subsp. *lactis*, *Latilactobacillus sakei*, verified by in vitro pathogenic bacteria inhibition and meat preservation verification	[119,122,123,124]
		lactocin S	Disrupt the cell membrane		
		lacticin 3147	Inhibits the biosynthesis of peptidoglycan; Disrupts the cell membrane.		
	Class II	pediocin PA-1	Disrupt the cell membrane, specific target Lipid II	*Pediococcus acidilactici*, verified through in vitro experiments	[125]
		pediocin-like bacteriocins		*Pentosaceus pediococcus*, verified through in vitro experiments	[126]
		enterocins L50A, L50B		*Enterococcus durans* EDD2, verified through in vitro experiments	[114]
		enterocin P			
		leuconocin S		*Weissella paramesenteroides*, verified through in vitro experiments	[116,127]
		enterocin AS-48	Internalized via vesicle-mediated endocytosis and induces autophagic cell death; specific target variant surface glycoprotein (VSG)	*Enterococcus faecalis*, verified by in vitro parasite inhibition experiments	[54]
		plantaricin W3-2	Disrupt the integrity of the cell membrane	*Lactiplantibacillus plantarum* W3-2, *Lactiplantibacillus plantarum* ZJ316, validated for the inhibition of foodborne pathogens in vitro	[128]
		plantaricin ZJ316			[122]
	Class III	helveticin J	Inhibits DNA replication, RNA synthesis, or protein synthesis	*Lactobacillus delbrueckii* subsp. *indicus* TY-11, validated for functional inhibition in vitro	[119,129]
		enterolysin A	Possesses enzymatic activity, causes cell lysis		
EPS		EPS of *L. plantarum* LRCC5310	High adhesiveness, interfering with the attachment of viruses to cells in vitro	Verified by in vitro antiviral experiments and intestinal cell	[130]
		EPS of *L. plantarum* YW32	Inhibits bacterial adhesion ability and interferes with biofilm activity		[63]
Others		CO2	Reduces the pH value of cells and the activity of enzymes; Generates an anoxic environment		[109]
		H2O2	Damages the molecular structure of proteins	*Lactobacillus bulgaricus*, *Lacticaseibacillus casei*, *L. lactis*, verified through experiments	[108,131]

### 4.2. Bacteriocin-Producing

Various bacteria could produce bacteriocins, among which LAB have been the most extensively studied. Bacteriocins are a class of bioactive precursor polypeptides, peptides, or proteinaceous antimicrobial substances, produced by certain bacteria (Table 3), which can interact with the cell surface, increase cell permeability, and inhibit cell wall synthesis, as well as nucleic acid and protein production (Figure 2) [132,133].

Bacteriocins produced by LAB can be classified into the following four categories: Class I bacteriocins are lanthipeptides, which are heat-stable and exhibit a broad antibacterial spectrum against Gram-positive bacteria, such as *Staphylococcus*, *Listeria*, and *Streptococcus* [134]. Based on structural differences, Class I bacteriocins are furtherly divided into type A, characterized by a positive charge, hydrophobic groups, amphiphilicity, and helical peptide chains; and type B, containing numerous small cyclic structures and with smaller molecular weights [134]. Class II bacteriocins are small, unmodified, heat-stable peptides that do not contain lanthionine, which are furtherly subdivided into four subclasses: IIa, IIb, IIc, and IId. Subclass IIa is the major group, demonstrating strong inhibitory activity against *Listeria monocytogenes*. Class III bacteriocins are heat-labile, large-molecular-weight proteins, of which the well-known nisin are the typical Class III bacteriocins produced by LAB (Figure S1). Compared to type A lanthipeptides, Class III bacteriocins exhibit a broader spectrum and more effective inhibition on both Gram-negative and Gram-positive bacteria. Class IV bacteriocins are large molecular complexes, whose active components include proteins, carbohydrates, lipids, etc. Class IV bacteriocins, the cyclic peptide substances, can effectively inhibit the growth of fungi and Gram-negative bacteria, but are rarely produced by LAB [135,136].

Bacteriocins produce by LAB are GRAS substances, and their application in the food industry has significantly increased due to their effective antimicrobial activity (Figure 2) [13]. The antimicrobial mechanism of bacteriocins is not singular. LAB-derived bacteriocins exert their antibacterial effects mainly through three distinct mechanisms. First and the most important, by targeting the cell membrane, class I bacteriocins (e.g., Nisin) bind to lipid II on the cytoplasmic membrane of the target bacteria, thereby not only inhibiting peptidoglycan synthesis but also forming transmembrane pores; class II bacteriocins (e.g., Pediocin PA-1 (Figure S1) bind to membrane receptors such as the mannose phosphotransferase system (Man-PTS) before assembling into pores, while some other bacteriocins non-specifically disrupt membrane integrity via electrostatic interactions. All these processes ultimately lead to the collapse of membrane potential and pH gradient, as well as the leakage of intracellular contents. Second, inhibiting cell wall synthesis, certain bacteriocins, including Nisin and Lantibiotic NAI-107, interfere with the functions of key components involved in cell wall biosynthesis, thus blocking peptidoglycan formation and causing bacterial death due to the failure to maintain cellular morphology against osmotic pressure. Third, interfering with intracellular metabolism and genetic materials, a subset of bacteriocins can penetrate into target bacterial cells, where they suppress bacterial proliferation by inhibiting DNA replication, RNA transcription, protein synthesis, or disrupting the cell cycle progression [133,137].

One bacteriocin may act with multiple inhibitory mechanisms, and the same bacteriocin may employ different mechanisms against different target cells. Unlike other inhibitory mechanism of action, AS-48 does not kill parasites by permeabilizing their plasma membrane. Instead, after interacting with the variant surface glycoprotein on the parasite surface, it is taken up clathrin-mediated endocytosis and induces autophagic cell death [54]. Reuterin is a special substance, produced by glycerol metabolism of *Limosilactobacillus reuteri*, and it exhibits diverse biological activities. Reuterin has a broad-spectrum inhibitory effect against both Gram-positive and Gram-negative bacteria. The antibacterial mechanism of Reuterin is to induce cellular oxidative stress by modifying the sulfhydryl groups in proteins and small molecules, and inhibit DNA synthesis, thereby suppressing bacterial growth [138].

Bacteriocins produced by LAB have high antimicrobial activity and broad inhibition spectrum. The cell-free supernatant of *Lactiplantibacillus plantarum* ST194BZ, containing bacteriocin ST194BZ could inhibit the growth of *Enterococcus faecalis*, *Escherichia coli*, *Enterobacter cloacae*, and *Pseudomonas aeruginosa*. The maximum total bacteriocin activity of 12 800 AU/mL was recorded cultivation 14 h in MRS broth [139]. Bacteriocins ST23LD and ST341LD, produced by *Lactiplantibacillus plantarum* strains ST23LD and ST341LD, respectively, with activities of 1460 AU/OD and 2850 AU/OD upon growth in MRS broth at an initial pH of 6.0 [140].

### 4.3. Competition of Niche, Nutrition and Ions

LAB can effectively inhibit the growth of harmful bacteria through direct and furious competitions: nutrient competition, niche competition, and the competition for special elements. By competition, LAB could efficiently reduce the infection of spoilage bacteria and pathogenic bacteria on plants, animals, and human beings (Figure 2).

Regarding niche competition, LAB can protect sites from invasion and colonization by pathogenic bacteria by preventing pathogens from adhering to the sites, inhibiting the growth of pathogenic bacteria. In the host, the prerequisite for pathogenic infection is the adhesion and colonization of the host intestine. LAB can adhere to epithelial cells via bonding between lipoteichoic acid on their cell surface and receptor sites on animal cell protein structures, thereby occupying colonization sites [141,142]. Simultaneously, LAB would alter the growth environment through metabolic products, for example, lower pH, furtherly reducing the infective ability of pathogens [143].

In terms of nutrient competition, the competition for nutrients is considered as an antimicrobial mechanism. Nutrition in certain environments is limited, and LAB rapidly consume essential nutrients such as carbohydrates, amino acids, and vitamins around the environment, leading to nutrient depletion. Subsequently, the harmful microorganisms have lower growth and abnormally reproduce, and, thus, are effectively suppressed [144]. LAB might efficiently use nutrients in medium when co-cultured with fungi, so that fungi cannot grow without certain essential nutrients. To investigate the key performance of nutrient competition, the stronger inhibition of *Lactobacillus kefiri* M4 on the growth of *Penicillium expansum* LPH10 in apple juice is examined [145]. It is revealed that upon the low nutrient concentration, LAB preferentially utilized the nutrients, resulting in suboptimal nutrient levels for the fungal germination [145].

In addition, several ions are essential for the normal growth of the microorganism, especially iron. Many bacteria secrete high-affinity siderophores to scavenge iron from the environment. Certain LAB strains also could produce their own unique siderophores, which intensely compete with other bacteria, strongly chelating free iron in the environment to form iron complexes [146]. As a result, harmful bacteria would suffer from severe iron deficiency, leading to metabolic arrest and growth inhibition [147]. In addition, metal ions also have a significant impact on the inhibitory activity of bacteriocins: on the one hand, metal ions can enhance the antibacterial effect of bacteriocins by altering the permeability of cell membranes; on the other hand, ions could change the spatial structure of bacteriocins, which leads to the variation in their antibacterial activity. Particularly, divalent metal ions exert a relatively strong influence on bacteriocins [148].

### 4.4. Extracellular Polymeric Substances (EPS) Production

EPS are a class of carbohydrate compounds secreted outside the cell wall during LAB growth and metabolism, which consists of repeating units of sugars or sugar derivatives, including glucose, galactose, and rhamnose in varying proportions (Table 3). EPS exists in two forms: capsular polysaccharides, which are structural components of the cell wall, and slime polysaccharides, which are released into the surrounding environment [149]. Based on differences in chemical composition, EPS can be furtherly classified into homopolysaccharides (HoPS), composed of a single type of monosaccharide, and HePS, composed of two or more types of monosaccharides [150]. Numerous studies have revealed that EPS exhibits various physiological functions and probiotic properties, such as immunomodulation, antioxidant activity, anti-ulcer effects, antitumor activity, improvement of intestinal microecology, and cholesterol-lowering effects, as well as antibacterial and antiviral actions [151,152]. Higher yields of EPS could enhance the niches competition and biofilm formation of LAB. Meanwhile, EPS can also improve the co-aggregation ability of LAB on other microorganisms, which reduce the colonization of harmful microorganisms [153]. Bajpai et al. found that the crude EPS extracts of *Lactiplantibacillus plantarum* 8513 exhibited excellent inhibitory activity against *Staphylococcus aureus* [153].

### 4.5. Quorum-Sensing (QS) Interference

Certain LAB strains exert antimicrobial effects by blocking the quorum-sensing (QS) signal pathway of pathogenic bacteria, subsequently inhibiting the growth of harmful microorganisms and biofilms (Figure 2). QS is a communication system among bacteria that mediates and coordinates collective behaviors in most bacteria, especially regulating physiological processes such as virulence factor expression, biofilm formation, pilus assembly, and secondary metabolite production [154]. Song et al. investigated the antifungal activity of various LAB against *Candida albicans*, and found the obvious inhibitory effects on biofilm formation of *Candida* [155]. Ni-Na et al. found that when *Levilactobacillus brevis* DF01 was co-incubated with *Escherichia coli* and *Salmonella typhimurium*, LAB strains could interfere with the biofilm formation of pathogenic bacteria, but could not destroy the already-formed biofilms, suggesting that *Levilactobacillus brevis* DF01 could interfere with the QS regulatory system of pathogens, and subsequently destroy their biofilm formation [156]. Kostoglou and Giaouris detected the inhibition of 89 CFS of LAB strains on QS systems with a luminescent reporter assay, which showed that the CFS of 32 LAB strains could inhibit the Acylated Hisostatin Lactone (AHL) system, while 38.2% of strains have interference on the Autoinducer-2 (AI-2) system [157]. Similarly, Rana et al. noticed that the cell-free supernatant of different LAB could inhibit the AHL level and AHL-related genes expression of *Pseudomonas aeruginosa* [75]. In the recent study, the research supposed that the secreted proteins and EPS of LAB might be the quorum quenching agents [158,159]. Although many studies have shown that LAB and their metabolites can interfere with the quorum-sensing regulatory systems of other microorganisms, the exact mechanism is still unclear. No specific metabolite of LAB has yet been identified as the quorum-quenching agent.

## 5. The Influences of LAB Inhibitory Activities

It is particularly noteworthy that the antimicrobial function of LAB is specific strain-dependent and environment-dependent property, regulating by a combination of intrinsic factors, such as genome constitution and gene expression, and extrinsic factors, including temperature, pH, nutrition, microorganism interaction, etc. Among these, nutrition composition, environment conditions, specific ion components, key signaling molecules, and intracellular regulatory factors collectively form an intricate regulatory network precisely modulating the inhibitory activity of LAB. Among these, nutrition composition, environment conditions, specific ion components, key signaling molecules, and intracellular regulatory factors collectively form an intricate regulatory network precisely modulating the inhibitory activity of LAB (Figure 1). Investigating the regulatory network could be significant theoretical and practical values for optimizing the antimicrobial potential of LAB, which would be promoting the applications of preservative LAB in the food industry.

### 5.1. Optimization of Growth Media

The compositions of media significantly influence the production of inhibitory metabolites of LAB. Several studies have been focused on the optimization of cultivation media to improve the inhibitory capacity of LAB. The carbon source of culture media is the most common regulatory factor. In the presence of 20 g/L glucose, maltose, mannose, or sucrose yielded bacteriocin levels of 6400 AU/mL, whereas the same concentration of lactose and fructose yielded only 3200 AU/mL and 1600 AU/mL, respectively [160]. Similarly, saline ions, especially phosphates, can also affect antimicrobial substance production. In a study, 20 g/L KH_2_PO_4_ enhanced bacteriocin ST34BR production achieving to 12,800 AU/mL [160]. Additionally, several Agro-products and food materials could be potential culture mediums constitution, with which LAB were fermented and showed an excellent antimicrobial effect. Jukonyte et al. found that using rice polish medium as the fermentation substrate for LAB, without adding extra hydrolase and nutrients, was comparable to MRS medium in regard to the inhibition of molds [161]. The study on the production of bacteriocin by *Lactiplantibacillus plantarum* ST194BZ found that adjustment of pH could not affect the bacteriocin production, but the addition of a combination of tryptone and meat extract (1:0.6) could significantly enhance the bacteriocin production, and in the presence of 30–40 g/L of mannose and 10–50 g/L of KH_2_PO_4_, the antimicrobial activities of LAB were almost doubled, with 25,600 AU/mL [162].

The yield of specific bacteriostatic compounds of LAB can also be increased by adding intermediate metabolites to the culture medium. PLA, an antifungal activity metabolite produced by LAB, productions of MRS broth was increased by three times after 24 h and by 4.2 times upon supplementing with 5% phenylalanine (Phe), reaching concentrations of 116.4 and 361.2 mg/L, respectively [163]. If phenylpyruvic acid (PPA), the precursor of PLA, was directly added into media, the PLA level of LAB was increased by 6-fold, and the antifungal activity was significantly improved consequently [164]. All these studies confirmed that, by specific modifications of the culture medium components, this effectively enhanced the antimicrobial activity of LAB and the yield of the specific antimicrobial compounds.

### 5.2. Culture Conditions

LAB would present the differential antimicrobial capacities at the changeable environment, and the activity of LAB also depends on cultural conditions in vitro, such as temperature, pH, rotation, incubation period, etc. Culture temperature and pH are two crucial environmental factors that regulate the antibacterial capacity of LAB and the yield of active substances. Suitable culture temperature and pH not only ensure efficient bacterial growth, but also serve as prerequisites for the synthesis of antimicrobial compounds. For the majority of LAB, the optimal temperature and optimal pH value is around 37 °C and pH 5.5–6.5, respectively [165,166]. However, the optimal combination of temperature and pH is the strain-specific character. For example, the optimal growth temperature of *Pediococcus acidilactici* GY317, a strain isolated from silage, is 40 °C, whereas the optimal growth conditions for *Pediococcus acidilactici* RSP-6, a strain separated from the intestinal tract of shrimp, are 35 °C and pH 6.0 [167,168]. But the optimal growth condition might not be suitable for demonstrating the inhibitory function of LAB, while some level of adverse situations would stimulate the antagonistic chemicals production of LAB. When the combination of temperature and pH was 37 °C and pH 6.5, respectively, the antifungal activity of *Lactiplantibacillus plantarum* K35 was up to its maximum after 48 h cultivation [169]. Similarly, the antifungal activity and antifungal metabolites production of *Lactiplantibacillus plantarum* CUK501 showed the peak upon 30 °C at the late logarithmic phase [170], and the lower initial pH was responsible for the highlighted antimicrobial phenotype of *Lactiplantibacillus plantarum* [171].

Beyond temperature and pH, the adjustment of cultivation agitation speed and gas composition has become a critical and precise control strategy for optimizing LAB fermentation processes, maximizing their biomass, antibacterial capacity, and the yield of active metabolites. The underlying rationale is that both agitation speed and gas environment collectively govern the dissolved oxygen level within the cultivation system. Different LAB may respond differently to oxygen, which some strains may enhance biomass under microaerophilic conditions, while others produce high yields of antibacterial substances only under strict anaerobic conditions. In the study by Arauz et al., the maximum nisin concentration produced by *Lactococcus lactis* in a 2 L bioreactor was found to be 49.88 mg/L (3.3 log AU/mL or 1995.29 AU/mL), achieved after 16 h of cultivation at 200 rpm without aeration [172]. According to the study by Wu et al., the optimized fermentation process successfully achieved high-density cultivation of Lactobacillus R8 by employing a lower agitation speed (100 rpm at laboratory scale and 60 rpm at pilot scale) in combination with intermittent nitrogen gas sparging to maintain an anaerobic environment [173]. Multiple studies have demonstrated that by adjusting culture conditions such as temperature, pH, agitation rate, and incubation period, we can actively steer the metabolism of LAB toward enhanced proliferation and efficient synthesis of target antimicrobial compounds.

### 5.3. Ionic Components in Cultural Condition

The antimicrobial function of LAB is influenced not only by macronutrients and environmental conditions, but is also finely regulated by specific ionic components and signaling molecules in the culture medium. Metal ions, serving as enzymatic cofactors or signaling molecules, extensively participate in the physiological metabolism of LAB and the synthesis of antibacterial substances.

Manganese ions (Mn^2+^), as cofactors for manganese superoxide dismutase (Mn-SOD), could help LAB resist oxidative stress and maintain the metabolic activity, indirectly ensuring the antagonistic characters [174]. Archibald and Fridovich concluded that *Lactiplantibacillus plantarum* has an unusually large requirement for Mn^2+^ ions [175]. And *Lactiplantibacillus plantarum* L73 strains exhibited a high demand for Mn^2+^ and could accumulate high levels of Mn^2+^ (over 30 mM) in cell. *Lactiplantibacillus plantarum* Mn^2+^, which function equivalently to the enzyme, to maintain metabolic activity and thereby ensure functional viability [175].

The effects of Ca^2+^ and Mg^2+^ on the antifungal activity of LAB isolates were also investigated. The Mg^2+^ ions act as cofactors for various phosphokinases and synthetases, participating in energy metabolism and nucleic acid synthesis [176]. MgSO_4_·7H_2_O (0.2 g/L) in diverse culture medium is essential for ensuring the maintenance of normal enzymatic activity [176]. Similarly, Ca^2+^ ions not only act as stabilizers of cell wall structure but also participate in signal transduction processes, influencing the physiological state of LAB [177]. Compared to natural conditions, the addition of Ca^2+^ stimulated antifungal effects of *Lactobacillus delbrueckii* subsp. *bulgaricus* against *Penicillium* spp., *Trichoderma viride*, and *Aspergillus flavus* [178]. The addition of Mg^2+^ induced antifungal activity in the combinations of four LAB isolates, which antifungal improvements were associated with the increasing of organic acid [178].

Notably, several LAB strains naturally exhibit tolerance to hyperosmotic conditions, enabling them to become the predominant microbiota and play a central role in high-salt preserved foods such as pickles and cheese [179]. The high osmotic pressure generated by elevated salt concentrations inherently inhibits the growth of most spoilage and pathogenic microorganisms, thereby exerting a preservative effect. Salt-tolerant LAB, through the evolution of efficient ion transport systems (e.g., K^+^ uptake, Na^+^/H^+^ antiporters), can maintain osmotic balance inside and outside the cells, allowing them not only to survive under high-salt stress but also to retain metabolic activity. For instance, some *Lactiplantibacillus plantarum* strains have been reported to be resistant to osmotic stress and capable of withstanding 50–120 g/L NaCl [179]. The *Pediococcus pentosaceus* strain HN10 was isolated from fermented foods, which achieved maximum growth rate at 50 g/L NaCl [180]. Appropriate concentrations of NaCl can stimulate certain LAB strains to more efficiently produce bacteriocins, organic acids, hydrogen peroxide, and other antimicrobial metabolites [181]. Building upon this adaptation, environmental salt and ionic strength serve as key factor regulating the antimicrobial activity of LAB.

### 5.4. Regulatory Signaling Molecules

Quorum Sensing (QS) is a crucial mechanism through which LAB perceive population density and coordinate group behaviors by secreting signaling molecules. The QS system of LAB could regulate the production of antagonistic metabolites such as EPS, conjugated linoleic acid, PLA, and antimicrobial peptides, which contribute to the antimicrobial properties of LAB [182].

The AI-2 signal molecule, serving as a “universal language” for interspecies communication, plays a key role in regulating the antibacterial function of LAB. The inhibitory effect of *Lactiplantibacillus pentosus* Z097 is significantly enhanced by the QS signal molecule Autoinducer-2 (AI-2) to *Listeria monocytogenes* [183]. LAB synthesize AI-2 by *luxS* gene, and LuxP and LsrB are the receptor of AI-2 signal, regulating the downstream gene expression, subsequently regulating a series of physiological phenotypes including bacteriocin synthesis, biofilm formation, and carbohydrate metabolism.

Furthermore, several studies have confirmed that in LAB, bacteriocin synthesis is regulated by the AIPs-two component (AIPs-TCS) system [184,185]. QS is activated when the bacterial cell density reaches a certain threshold, while upon inoculation at a very low cell density, some LAB strains use a AIPs-TCS QS system controlling their bacteriocin-producing capacity [186]. The productions of bacteriocin of LAB were enhanced when the unique AIP factor PLNC8IF was added to the culture [187]. All information is indicated that exposure to the specific QS signal molecules can also induce bacteriocin synthesis without requiring potentially costly and risky procedures. Understanding this regulatory network is of great significance for the targeted modulation of the antimicrobial function of LAB.

### 5.5. Key Regulatory Factors

The antimicrobial functions of LAB are ultimately controlled by various regulatory factors in their genomes. Different factors receive internal and external signals, coordinate the metabolic allocation, and determine the timing and production of antimicrobial substance. In LAB, the typical bacteriocin biosynthesis clusters would be including structural genes, processing genes, tolerance genes, and transport genes, as well as the cluster regulatory genes. Among these, transcriptional regulatory factors act as the central molecular switches and directly control the timing and level of bacteriocin synthetic gene transcription [188]. For bacteriocin biosynthesis, upon the extracellular signal molecules accumulating to the threshold, sensor kinases are activated, triggering a phosphorylation cascade, which activates the cluster inducer regulators, and subsequently initiates the bacteriocin synthesis genes transcription. In contrast, the repressors would block the expression of bacteriocin genes under the unnecessary conditions, avoiding energy waste [188]. In studies of *Lactococcus lactis*, autoinducers, such as Nisin, activate the quorum-sensing system, promoting the synthesis of nisin, thereby enhancing antibacterial activity within the population. Two genes in the cluster, *nisA* and *nisF*, are induced by nisin via a two-component signal transduction pathway consisting of a histidine protein kinase, *NisK*, and a response regulator, *NisR*. Transcriptional expressions of both *nisR* and *nisK* are driven from the constitutive promoter of NisR [189]. It has been reported that a two-plasmid system in which the *nisA* promoter and the regulatory genes *nisR* and *nisK* are used allows efficient control in a variety of LAB [190].

Numerous global regulators are also involved in modulating the synthesis of antimicrobial substances and the antibacterial activity of LAB. PprI is a global regulatory factor in LAB, and improvement its expression can increase lactic acid production and stress resistance of LAB [191]. Two-component systems such as VicR-K (regulatory factor VicR and kinase VicK), ComD-E (regulatory factor ComE and kinase ComD), and CiaR-H (regulatory factor CiaR and kinase CiaH) could regulate multiple metabolic processes in LAB, including organic acid production, EPS formation, and antimicrobial substances synthesis [185]. Accordingly, optimization of antimicrobial substance gene expression would help the development of LAB as microbial cell factories for production and delivery of potential antibiotics of biotechnological interest.

## 6. Conclusions and Prospect

The demand of minimally processed and more natural foods is continuously growing. Finding the natural and safe alternatives to chemical preservatives has been investigated and raised for some time now. There is a trend with biological preservation to control the spoiling of food. LAB, as an important group of probiotics, could inhibit various foodborne pathogens and spoilage microorganisms with various weapons, including organic acids, bacteriocins, and hydrogen peroxide, as well as competitive exclusion for living space and nutrition. LAB and its antimicrobial metabolites would be better applicated in food preservation, which are not only efficient and safe, but also remain active in complex food matrices (Figure 2).

However, the large-scale application of LAB and their metabolites as preservatives in the food industry still face numerous challenges and inherent limitations. First, their performance is unstable: most antimicrobial metabolites are sensitive to environmental factors such as temperature and pH, prone to degradation and inactivation during processing and storage, and their antimicrobial efficacy can be further impaired by interactions with components in complex food matrices. Second, the natural yield of antimicrobial substances is low; fermentation optimization and the complex processes involved in metabolite extraction and purification lead to high production costs, making them difficult to compete with chemical preservatives. Third, large-scale fermentation is susceptible to various factors, resulting in unstable batch-to-batch yield and activity of products, difficulty in ensuring strain consistency, and great challenges in quality control. Fourth, the antimicrobial activity of LAB strains is highly specific, leading to limited applicability; in addition, some strains may produce harmful metabolites or carry antibiotic resistance genes, posing potential safety risks. Fifth, they have insufficient compatibility with existing processing systems, which may affect the sensory quality of food products and make it difficult to directly integrate them into traditional production lines [192,193]. So, there are still many issues that need to be resolved in order to truly substitution of chemical preservatives with antimicrobial LAB or their metabolites.

With deepening investigations of the antagonistic features and mechanisms of LAB, various strategies to optimize and enhance their antimicrobial performance in food have been employed. By screening for novel functional strains, optimizing fermentation conditions, directed inducing and gene editing for LAB, developing the novel delivery systems, as well as constructing cell factories of antimicrobial metabolites, diverse efforts not only have promoted the inhibitory capacities of LAB, but also have modified LAB suitable for food preservation, providing new insights of novel antimicrobial agents (Figure 1).

### 6.1. Screening of Novel Probiotics and Exploration of Novel Functions of LAB

Isolation remains an important method for obtaining the novel active LAB. However, traditional isolation methods have been faced the bottleneck. More than 80% of microorganisms in nature cannot be isolated and cultured by routine methods and media [194]. Microbial culturomics technologies and novel cultural conditions would be contributed to find new LAB isolates. Lagier et al. [195] identified the human gut microbiome using culturomics, and discovered 174 previously undescribed species, including 31 novel species and genera. By modulating the nutritional components, gas conditions, and physicochemical parameters, previously unculturable LAB could be cultivated by researchers now, cultivate species, mining more inhibitory LAB strains in future.

Previous studies have investigated the inhibitory capabilities of LAB by antagonistic tests and inhibitory substances analysis. With the development of sequencing technology, the antimicrobial relevant genes would be noticed by whole genomic and metagenomic analyses, subsequently, the underlying antagonistic LAB strains would be found. Mkadem et al., by going through whole genome sequencing and analysis of genes encoding bacteriocins, found that genes encoding bacteriocin production were only detected in *Lacticaseibacillus paracasei* OWS23 for LSEI 2386 and bacteriocin immunity protein, which is speculated that *Lacticaseibacillus paracasei* OWS23 have the ability to inhibit the growth of *Listeria* spp. [196]. Consequently, there is an urgent need for genetic differentiation of strains with strong antifungal activity to clearly define all possible strains within a species and their corresponding antifungal spectra. By integrating omics technologies such as genomics, transcriptomics, and metabolomics, combined with bioinformatics predictions, it provides a powerful potential for discovering novel antibacterial functions and compounds of LAB. Although omics technologies have significantly advanced the mining of functional LAB, there are still some technical bottlenecks that need to be addressed. In metabolomics, some compounds are difficult to detect due to concentrations falling below the detection limit of instruments. It should be emphasized that the diversity and variety of antifungal molecules identified in LAB to date make their exhaustive identification and quantification challenging. Therefore, future research should focus on constructing a big data platform for the antibacterial functions of LAB. This platform would integrate genomic, transcriptomic, proteomic, and metabolomic data, utilize artificial intelligence algorithms to mine key functional marker genes, and establish predictive models for antibacterial phenotypes.

### 6.2. AI-Driven Optimization of Culture Media and Culture Conditions

Due to the differences in genome information and metabolic modes among different species of LAB, their requirements for nutrients and adaptability to external environments vary. Therefore, personalized adjustment of medium formulations and cultivation condition can effectively increase the cell density and the yield of functional metabolites. However, numerous conditions would affect the fermentation performance of LAB, such as carbon sources, nitrogen sources, inorganic salts, growth factors, temperature, pH, and feeding strategies. These factors interact with each other, making the selection of optimal culture and fermentation conditions a complex and challenging research topic.

The traditional primary method for optimizing conditions is the combination of single-factor experiments and response surface methodology (RSM). RSM exhibits significant superiority in experimental design, model construction, and independent variable evaluation [197]. Nevertheless, this method still has the drawback of an enormous workload. As an interdisciplinary field, artificial intelligence (AI) technology mainly includes machine learning (ML), deep learning, artificial neural networks (ANN), genetic algorithms (GA), and fuzzy logic (FL) [198]. Compared with RSM, ML and ANN may be more suitable for optimizing the medium formulations and cultivation condition of certain LAB strains [198,199]. Wang et al. [199] established an intelligent hybrid model combining ANN and GA (ANN-GA), to optimize the medium components of *Lactiplantibacillus plantarum*. The results showed that the predicted optimal biomass of *Lactiplantibacillus plantarum* was in close agreement with the experimental values under optimized conditions, indicating that the trained ANN-GA model had excellent predictive and optimization capabilities [199].

To overcome the adverse effects of LAB metabolites on fermentation, methods such as chemical neutralization, fed-batch culture and dialysis culture are often used to alleviate the impacts of LAB metabolites. Yet these methods also suffer from disadvantages including high costs and unsatisfactory efficacy. Sorensen et al. established an ANN-based model on the basis of exponential feeding strategy, and dynamically adjusted the feeding rate to sustain the growth of *Lacticaseibacillus rhamnosus* [200]. The findings demonstrated that ANN-driven dynamic feeding control was more precise than traditional fed-batch culture [200]. The application of AI-driven technical approaches to further optimize fed-batch culture modes will facilitate the realization of high-density culture of LAB and efficient acquisition of fermentation products in modern fermentation technology.

### 6.3. Strain Modification with Induced Mutation and Gene Editing

Modifying the original LAB genomic information is to enhance their probiotic properties, fermentation efficiency, or to confer new functions, mainly divided into traditional breeding and modern genetic engineering methods.

Ultraviolet mutagenesis, irradiation mutagenesis, and space mutagenesis are proven breeding techniques that alter the DNA structure of LAB. In a study by Goodarzi et al., it was found that the yield of rifampicin- and streptomycin-resistant mutants of LABs depending of UV exposure [201]. Shan et al. found that three highly effective acid-reducing LAB from the irradiated sauerkraut X, sequencing as *Pediococcus ethanolidurans*, *Levilactobacillus brevis*, and *Loigolactobacillus coryniformis* [202]. Furthermore, exposure LAB to the space environment, *Lacticaseibacillus rhamnosus* Probio-M9 not only exhibits favorable growth characteristics, but also demonstrates excellent probiotic properties [203].

Gene recombination technology enables LAB to acquire new genetic traits by introducing foreign DNA fragments. The inhibitory activities of LAB strains F3C2 and F3A3 were improved to 192% and 200%, respectively, after genome shuffling with *Lactiplantibacillus plantarum* IMAU10014 and *Lactobacillus helveticus* IMAU40097 [204]. Another study found that the co-cultivation of LABs could lead to changed antagonistic activity, which is significant for the creation and further investigation of LAB associations as antifungal agents.

With the rapid development of gene editing technologies like CRISPR, the rational design of LAB has entered a new stage. Precise regulation of specific gene expression can significantly enhance their antibacterial activity and broaden their antibacterial spectrum. Son et al. developed a CRISPRi platform in *Leuconostoc citreum*, which can be used for simultaneously downregulating the expression of *ribF* and *folE* genes, thereby improving riboflavin production [205]. Compared to traditional mutagenesis techniques, gene editing offers advantages of precision and high efficiency, providing a powerful tool for constructing high-performance engineered strains. Despite the promising prospects of gene editing, the biosafety of engineered strains and public acceptance remain key factors restricting their application in food. Therefore, it is necessary to implement appropriate experimental methodologies to select bacteriocin-producing strains suitable for food production.

Heterologous expression of bacteriocin genes can improve strain characteristics and bacteriocin production yields, and the timing of bacteriocin production can be adjusted using inducible production systems, which opens possibilities for further research into the potential for LAB to overproduce metabolites.

### 6.4. Nano-Encapsulation and Active Packaging Technology

The encapsulation and delivery of LAB or their antibacterial metabolites using nanotechnology is an important strategy for improving their stability and application efficacy. Nano-encapsulation technology offers significant advantages over traditional direct addition methods. Encapsulation technology can enable controlled release of LAB at specific sites, protect LAB and their metabolites from degradation by oxygen, heat, and gastric acid, and the nanoscale particles can interact more easily with target microorganisms, potentially enhancing bioavailability [206]. Bioactive substances such as bacteriocins, organic acids, and EPS produced by LAB often have application bottleneck such as poor stability, easy degradation, and low absorption and utilization rate in the body [207]. Nano-encapsulation technology can significantly improve their stability and shelf life, and greatly enhance bioavailability and efficacy [207]. Nanotechnology can also enable the synergistic delivery of LAB and other active compounds, achieving their co-encapsulation and protection, effectively enhancing their biological properties. In a study by Dawwam et al., bacterial cellulose (BC) synthesized by *Limosilactobacillus fermentum* 6BC and zinc oxide (ZnO) nanoparticles were integratedly constructed a multifunctional bioactive platform capable of simultaneously loading curcumin and propolis extract, exhibiting antibacterial, antioxidant wound-healing properties, and maintaining excellent biocompatibility [208]. Zhang et al. used *Lacticaseibacillus rhamnosus* “bacterial ghosts” (LBGs) as a natural biomimetic carrier to construct an astaxanthin delivery system with dual targeting for the liver and mitochondria, which can not only effectively overcome the bioavailability limitations of astaxanthin, but also achieve precise accumulation of the drug at liver lesion sites, providing a new approach for targeted intervention in non-alcoholic steatohepatitis [209]. This precise co-delivery system shows great potential in functional foods, oral vaccines and disease treatment, enhancing intervention effectiveness, as well as laying the foundation for the development of the next generation of smart delivery systems.

Active packaging materials based on nano-encapsulation represent another innovative direction. The application of LAB and their metabolites in the field of active packaging focuses on three directions: antibacterial effect improvement, edible film/coating construction, and intelligent controlled release. Embedding LAB antibacterial substances into packaging film matrices allows for the slow release of active components throughout the food shelf life, providing continuous protection. The carriers of such active packaging are mostly degradable biopolymers, including cellulose, chitosan, and alginate, which are fabricated via processes such as impregnation, blending, and microencapsulation [210]. After adding 2% *Lactobacillus* to the edible films used for food packaging, the antioxidant activity of the films was significantly enhanced, and the shelf life of the film packaging materials and the packaged food were effectively prolonged [211]. To further enhance the application efficiency, encapsulation and immobilization technologies are commonly adopted in the industry to improve the storage stability and processing tolerance of active ingredients. Four cyclic dipeptides from *Lacticaseibacillus paracasei* ZX1231 were optimally combined with a bacterial nanocellulose matrix to prepare the active quality packaging films, significantly inhibiting the fungi growth and thus prolonging the shelf life of bread, beef, cheese, and soy sauce [212]. However, this field is still confronted with several practical challenges, such as the easy inactivation of active ingredients during processing, the high purification cost of some highly active metabolites, and the inconsistent management and control standards across different regions [213]. In the future, relevant research will focus on optimizing the screening of LAB strains and metabolic regulation to improve antibacterial activity, developing pH/temperature-responsive intelligent release systems to achieve precise preservation, and expanding the application scenarios of LAB-based active packaging in more food categories, so as to promote its large-scale and commercial development.

### 6.5. Development of LAB as Expression Vectors and Cell Factories

The core advantages of LAB as a cellular factory lie in their high safety (GRAS certification), a mature genetic manipulation system that facilitates modification, and efficient fermentation characteristics such as the ability to utilize inexpensive raw materials, rapid growth, and often anaerobic fermentation. They can also secrete target products directly extracellularly, greatly simplifying subsequent separation and purification processes. These features make them a safe, efficient, and cost-effective microbial production platform in the fields of food, medicine, and other sectors. Ribeiro et al. used the pWV01 and pAMβ1 plasmids to express the Brucella abortus antigen L7/L12 in *Lactococcus lactis* [214]. When the protein was expressed in the cytoplasm, the maximum yield reached 0.5 mg·L^−1^, whereas the yield from secretion could reach 3.0 mg/L [214]. To ward against H1N1 virus infection, *Lactiplantibacillus plantarum* ZN3 was genetically engineered to express a surface-displayed fusion protein, containing the HA1 protein, DC-targeting peptide, and M cell-targeting peptide, which could induce effective immune responses in the intestine and upper respiratory airways, thus increasing the survival rate of mice [215].

As microbial cell factories, LAB have significant potential and unique advantages. However, this system still faces multiple challenges: (1) its relatively limited metabolic network and regulatory tools result in inadequate capacity for synthesizing complex compounds; (2) the introduction of exogenous pathways often causes excessive metabolic burden, affecting bacterial growth and product yield; (3) gene editing efficiency is relatively low and the precision of expression regulation needs improvement; (4) issues such as optimization of cultivation in industrial-scale fermentation, product extraction, and cost control also need to be addressed.

Using LAB for bio-preservation is a major trend. Currently, the large-scale application of LAB faces numerous challenges. To address these issues, strategies such as screening for new strains, optimizing the fermentation process, genetic engineering, and developing nano-delivery systems are being employed. The prospects are vast and diverse. With the ongoing development of synthetic biology and food safety assessment systems, lactic acid bacteria are expected to provide the next generation of green preservation solutions, thereby contributing to safer, healthier, and more sustainable food systems.

## Figures and Tables

**Table 2:** The inhibition/removal of LAB on harmful microbes and toxic substances.

Foodborne Harmful Substance		Suppress/Removal Target	Lactic Acid Bacteria	Suppress/Removal Effects	References
Fungi		*Penicillium digitatum*	*Lactiplantibacillus plantarum*	Washed cell suspension, 9% to 100% inhibition; CFS, 99.4% to 100% inhibition	[42]
			*Lactobacillus parafarraginis*	Wash cell suspension, 35.4% to 100% inhibition; CFS, 92.5% to 97.1% inhibition	
			*Lacticaseibacillus casei*	CFS, 92.5% to 97.1% inhibition	
			*Lacticaseibacillus paracasei* SMSP2	100% inhibition	
		*Aspergillus flavus*	*Lactiplantibacillus plantarum* 5L1	2.4 log units reduction	[38]
		*Penicillium verruculosum*			
		*Aspergillus carbonarius* ISPA 5010	*Lactiplantibacillus plantarum* E3	MIC, 25 g·L^−1^; MFC, 50 g·L^−1^	[43]
		*Aspergillus ochraceus* CECT 2093		MIC, 12.5 g·L^−1^; MFC, 100 g·L^−1^	
		*Botrytis cinerea* CECT 20973		MIC, 6.3 g·L^−1^; MFC, 6.3 g·L^−1^	
		*Aspergillus niger* CECT 2915		MIC, 25 g·L^−1^; MFC, 100 g·L^−1^	
		*Aspergillus tubingensis* CECT 20543		MIC, 25 g·L^−1^; MFC, 50 g·L^−1^	
Bacteria	Foodborne pathogens	*Listeria monocytogenes* LMG10470	*Lactiplantibacillus plantarum* LPS10	CFS, 100% inhibition; NCFS, 50% inhibition;	[44]
			*Limosilactobacillus fermentum* PP17	CFS, 56% inhibition; NCFS, 25% inhibition;	
			*Pediococcus acidilactici* MH512904	CFS, 75% inhibition; NCFS, 50% inhibition; 100% inhibition in pickled olives;	
		*Staphylococcus aureus* ATCC25923	*Lactiplantibacillus plantarum* LPS10	CFS, 100% inhibition; NCFS, 55% inhibition; 100% inhibition in pickled olives;	
			*Limosilactobacillus fermentum* PP17	CFS, 100% inhibition; NCFS, 55% inhibition;	
			*Pediococcus acidilactici* MH512904	CFS, 44.4% inhibition; NCFS, 12.5% inhibition; 100% inhibition in pickled olives;	
		*Bacillus cereus* ATCC14579	*Lactiplantibacillus plantarum* LPS10	CFS, 50% inhibition; NCFS, 55.6% inhibition;	
			*Limosilactobacillus fermentum* PP17	CFS, 50% inhibition; NCFS, 25% inhibition;	
			*Pediococcus acidilactici* MH512904	CFS, 75% inhibition; NCFS, 12.5% inhibition; 100% inhibition in pickled olives;	
		*Escherichia coli* ATCC25922	*Lactiplantibacillus plantarum* LPS10	CFS, 40% inhibition; NCFS, 6% inhibition; 100% inhibition in pickled olives;	
			*Limosilactobacillus fermentum* PP17	CFS, 37.5% inhibition; NCFS, 10% inhibition;100% inhibition in pickled olives;	
			*Pediococcus acidilactici* MH512904	CFS, 87.5% inhibition; NCFS, 25% inhibition; in pickled olives; 100% inhibition in pickled olives;	
		*Listeria monocytogenes* NICPBP 54002	*Lactiplantibacillus pentosus* 31-1	26% inhibition	[21]
		*Salmonella enteritidis* KCCM 12021	*Latilactobacillus curvatus* KCCM 43119	CFS, 100% inhibition; NCFS, 41% inhibition;	[45]
			*Leuconostoc mesenteroides* KCCM 43060	CFS, 96% inhibition; NCFS, 24% inhibition;	
			*Weissella cibaria* KCTC 3746	CFS, 97% inhibition; NCFS, 7% inhibition;	
			*Weissella koreensis* KCCM 41517	CFS, 97% inhibition; NCFS, 25% inhibition;	
		*Staphylococcus aureus* KCCM 11335	*Latilactobacillus curvatus* KCCM 43119	CFS, 98% inhibition; NCFS, 36% inhibition;	
			*Leuconostoc mesenteroides* KCCM 43060	CFS, 99% inhibition; NCFS, 10% inhibition;	
			*Weissella cibaria* KCTC 3746	CFS, 98% inhibition; NCFS, 26% inhibition;	
			*Weissella koreensis* KCCM 41517	CFS, 99% inhibition; NCFS, 33% inhibition;	
		*Salmonella typhimurium* KCTC 1925	*Latilactobacillus curvatus* KCCM 43119	CFS, 95% inhibition; NCFS, 46% inhibition;	
			*Leuconostoc mesenteroides* KCCM 43060	CFS, 96% inhibition; NCFS, 51% inhibition;	
			*Weissella cibaria* KCTC 3746	CFS, 98% inhibition; NCFS, 58% inhibition;	
			*Weissella koreensis* KCCM 41517	CFS, 97% inhibition; NCFS, 54% inhibition;	
		*Escherichia coli* O157:H7 ATCC 35150	*Latilactobacillus curvatus* KCCM 43119	CFS, 93% inhibition; NCFS, 34% inhibition;	
			*Leuconostoc mesenteroides* KCCM 43060	CFS, 97% inhibition; NCFS, 19% inhibition;	
			*Weissella cibaria* KCTC 3746	CFS, 100% inhibition; NCFS, 35% inhibition;	
			*Weissella koreensis* KCCM 41517	CFS, 100% inhibition; NCFS, 36% inhibition;	
		*Escherichia coli* MTCC 118	*Lacticaseibacillus paracasei* subsp. tolerans N2	73.3% inhibition;	[22]
			*Lacticaseibacillus casei* subsp. casei TM1B		
	Spoilage microbes	*Pseudomonas fluorescens* CGMCC 1.55	*Lactiplantibacillus pentosus* 31-1	20% inhibition	[21]
		*Brochothrix thermosphacta*	*Latilactobacillus curvatus*	4.5% inhibition	[46]
		*Brochothrix thermosphacta*	*Latilactobacillus sakei*	28.1% inhibition	
		*Enterbacteriaceae*	*Latilactobacillus curvatus*	12.3% inhibition	
		*Enterbacteriaceae*	*Latilactobacillus sakei*	35% inhibition	
		*Pseudomonas* spp.	*Latilactobacillus curvatus*	6.5% inhibition	
			*Latilactobacillus sakei*	15.5% inhibition	
		*Pseudomonas aeruginosa* MTCC 1934	*Lacticaseibacillus paracasei* subsp. *tolerans* N2	64.7% inhibition	[22]
		*Pseudomonas aeruginosa* MTCC 1934	*Lacticaseibacillus casei* subsp. *casei* TM1B		
		*Brochothrix thermosphacta*	*Latilactobacillus sakei*	58.2% inhibition	[47]
		*Pseudomonas* spp.	*Latilactobacillus sakei*	10.4% inhibition	
		*Enterbacteriaceae*	*Latilactobacillus sakei*	19% inhibition	
		*Pseudomonas fragi*	*Latilactobacillus sakei*	99.9% inhibition	[48]
			*Latilactobacillus sakei* CECT 4808	99.9% inhibition	
			*Lactobacillus* spp.	Uninhibited	
			*Lacticaseibacillus rhamnosus*	99.9% inhibition	
Virus		influenza A virus (A/NWS/33, H1N1 subtype)	*Enterococcus faecalis* KH2	62.5~88.2% inhibition	[49]
		Murine norovirus S99	*Latilactobacillus curvatus*	1.25 log units reduction	[50]
		Feline calicivirus (FCV)	*Lactococcus lactis* subsp. LM0230	1.8 log units reduction	[51]
		Rotaviruses	*Lactiplantibacillus plantarum* LRCC5310	41.3% inhibition	[52]
Foodborne parasites		*Trypanosoma cruzi* SN3 (IRHOD/CO/2008/SN3)	*Enterococcus faecalis* UGRA10	IC50, 0.11~6.81 μM	[53]
		*Trypanosoma cruzi* Arequipa (MHOM/Pe/2011/Arequipa)		IC50, 0.17~0.99 μM	
		*Trypanosoma cruzi* Tulahuen (TINF/CH/1956/Tulahuen)		IC50, 0.19~1.98 μM	
		*Trypanosoma brucei rhodesiense*		EC50, 1.70 ± 0.19 nM	[54]
		*Trypanosoma brucei gambiense*		EC50, 3.12 ± 0.15 nM	
		*Trypanosoma brucei brucei*		EC50, 2.61 ± 0.08 nM	
		*Leishmania donovani*		IC50, 3.9 ± 1.1~19.5 ± 2.1 μM	[55]
		*Trichinella*	*Levilactobacillus brevis* PQ214320	91% ~96% inhibition	[56]
Hazard Materials	Mycotoxins	Aflatoxin B1 (AFB1)	*Lactiplantibacillus plantarum* B3	27% removal	[57]
		Ochratoxin A (OTA)		32% removal	
		Zearalenone	*Lactobacillus acidophilus*	90.39% removal	[58]
			*Lactiplantibacillus plantarum* L1	88.68% removal	
			*Lactiplantibacillus plantarum* L2	86.49% removal	
			*Lacticaseibacillus paracasei*	86.14% removal	
		Acrylamide	*Lactiplantibacillus plantarum*	45–55% removal	[59]
	Heavy metals	Pb	*Levilactobacillus brevis*	adsorption 43.26 mg/g	[60]
			*Lactiplantibacillus plantarum*	adsorption 54.03 mg/g	
		Cd	Weissella viridescens ZY-6	<10 mg/L Cd^2+^, 100% removal; <100 mg/L Cd^2+^, 40% removal	[61]
		Pb, Cd, Ni	*Lacticaseibacillus casei*	47.65% Pb; 24.87% Cd; 67.72% Ni	[62]
			*Lacticaseibacillus rhamnosus*	62.35% Pb; 49.74% Cd; 78.42% Ni	
			*Lactiplantibacillus plantarum*	66.60% Pb; 53.06%; 81.82% Ni	
			*Limosilactobacillus fermentum*	76.59% Pb; 52.60% Cd; 81.53% Ni	
			*Enterococcus faecium*	79.75% Pb; 75.28% Cd; 83.99% Ni	
			*Lactobacillus helveticus*	50.70% Pb; 22.27% Cd; 78.99% Ni	
			*Lactobacillus acidophilus*	55.62% Pb; 48.34% Cd; 76.21% Ni	
	Pesticide	Organophosphorus pesticides (OPPs)	*Lactobacillus acidophilus* CICC20244	50 mg/L OPPs, 18% removal; 250 mg/L OPPs, 10% removal	[63]
			*Limosilactobacillus reuteri* CICC23151	50 mg/L OPPs, 12% removal; 250 mg/L OPPs, 15% removal	
			*Lactiplantibacillus plantarum* CICC20261	50 mg/L OPPs, 83% removal; 250 mg/L OPPs, 52% removal	
			*Bifidobacterium animalis* CICC21717	50 mg/L OPPs, 51% removal; 250 mg/L OPPs, 12% removal	
			*Lactobacillus helveticus* CICC6032	50 mg/L OPPs, 16% removal; 250 mg/L OPPs, 15% removal	
			*Lactobacillus delbrueckii* subsp. *bulgaricus* CICC6047	50 mg/L OPPs, 23% removal; 250 mg/L OPPs, 4% removal	

## Data Availability

The original contributions presented in this study are included in the article/Supplementary Materials. Further inquiries can be directed to the corresponding author.

## Supplementary Materials

The following supporting information can be downloaded at: https://www.mdpi.com/article/10.3390/foods15020241/s1, Figure S1: Chemical structures of antimicrobial metabolites of lactic acid bacteria.
