# Supplementary material for: Lactic Acid Bacteria as the Green and Safe Food Preservatives: Their Mechanisms, Applications and Prospects
Source: Foods. 2026 Jan 9;15(2):241. doi: 10.3390/foods15020241 (PMC12839946; doi:10.3390/foods15020241)
Supplement: Supplementary file 1 [file foods-15-00241-s001.zip › foods-4073234-supplementary.pdf]

Figure S1. Chemical structures of antimicrobial metabolites of lactic acid bacteria.

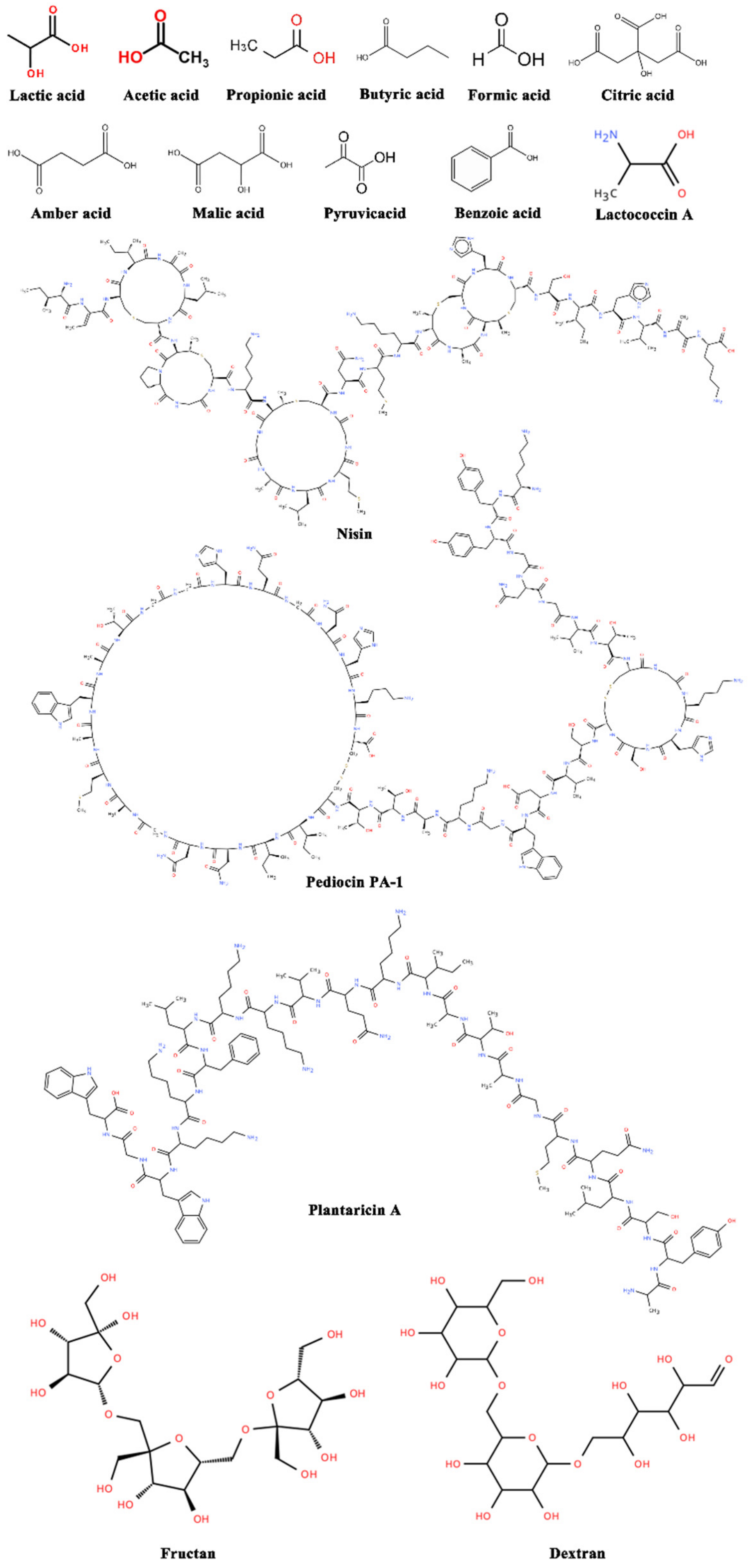

Several chemical structures of antimicrobial secondary metabolites from LAB, such as organic acid (Lactic acid, Acetic acid, Propionic acid, etc.), bacteriocin (Lactococcin A, Nisin, Pedocin PA-1, and Plantaricin A), and exopolysaccharides (Fructan and Dextran).
